# Supplementary material for: Five Unreported Ketone Compounds—Penicrustones A–E—From the Endophytic Fungus Penicillium crustosum
Source: Microorganisms. 2024 Oct 30;12(11):2195. doi: 10.3390/microorganisms12112195 (PMC11596421; doi:10.3390/microorganisms12112195)
Supplement: Supplementary file 1 [file microorganisms-12-02195-s001.zip › microorganisms-3254348-supplementary.pdf]

Supporting Information

Five unreported ketone compounds, penicrustones A-E from the endophytic fungus *Penicillium crustosum*

Dongmei Lin[1], Lian Yang[1], Jin Yang[1], Feixing Li[1], Xiuming Cui[1,2], Xiaoyan Yang[1,2,*]

[1] Faculty of Life Science and Technology, Kunming University of Science and Technology, Kunming 650500, China;

[2] Yunnan Key Laboratory of Sustainable Utilization of *Panax Notoginseng*, Kunming 650500, China

* Correspondence: yangxy@kust.edu.cn (X. Y.).

Tables of Contents

Figure S1. ^{1}H NMR spectrum (600MHz, $CDCl_3$) of **1** 4

Figure S2. ^{13}C spectrum (150MHz, $CDCl_3$) of **1** 4

Figure S3. DEPT spectrum of **1** 5

Figure S4. HSQC spectrum of **1** 5

Figure S5. ^{1}H-^{1}H COSY spectrum of **1** 6

Figure S6. HMBC spectrum of **1** 6

Figure S7. HR-ESI-MS spectrum of compound **1** 7

Figure S8. IR spectrum of compound **1** 8

Figure S9. UV spectrum of compound **1** 8

Figure S10. ^{1}H NMR spectrum (600MHz, $CDCl_3$) of **2** 9

Figure S11. ^{13}C spectrum (150MHz, $CDCl_3$) of **2** 9

Figure S12. DEPT spectrum (150MHz, $CDCl_3$) of **2** 10

Figure S13. HSQC spectrum of **2** 10

Figure S14. ^{1}H-^{1}H COSY spectrum of **2** 11

Figure S15. HMBC spectrum of **2** 11

Figure S16. HR-ESI-MS spectrum of compound **2** 12

Figure S17. IR spectrum of compound **2** 13

Figure S18. UV spectrum of compound **2** 13

Figure S19. ^{1}H NMR spectrum (600MHz, $CDCl_3$) of **3** 14

Figure S20. ^{13}C spectrum (150MHz, $CDCl_3$) of **3** 14

Figure S21. DEPT spectrum (150MHz, $CDCl_3$) of **3** 15

Figure S22. HSQC spectrum of **3** 15

Figure S23. ^{1}H-^{1}H COSY spectrum of **3** 16

Figure S24. HMBC spectrum of **3** 16

Figure S25. HR-ESI-MS spectrum of compound **3** 17

Figure S26. IR spectrum of compound **3** 18

Figure S27. UV spectrum of compound **3** 18

Figure S28. CD spectra of compound **3** 19

Figure S29. ^{1}H NMR spectrum (600MHz, $CDCl_3$) of **4** 19

Figure S30. ^{13}C spectrum (150MHz, $CDCl_3$) of **4** 20

Figure S31. HSQC spectrum of **4** 20

Figure S32. ^{1}H-^{1}H COSY spectrum of **4** 21

Figure S33. HMBC spectrum of **4** 21
Figure S34. HR-ESI-MS spectrum of compound **4** 22
Figure S35. IR spectrum of compound **4** 23
Figure S36. UV spectrum of compound **4** 23
Figure S37. ^{1}H NMR spectrum (600MHz, $CDCl_3$) of **5** 24
Figure S38. ^{13}C spectrum (150MHz, $CDCl_3$) of **5** 24
Figure S39. DEPT spectrum (150MHz, $CDCl_3$) of **5** 25
Figure S40. HSQC spectrum of **5** 25
Figure S41. ^{1}H-^{1}H COSY spectrum of **5** 26
Figure S42. HMBC spectrum of **5** 26
Figure S43. HR-ESI-MS spectrum of compound **5** 27
Figure S44. IR spectrum of compound **5** 28
Figure S45. UV spectrum of compound **5** 28
Figure S46. Chiral analysis and preparation of **(±)-1** 29
Figure S47. Initial evaluation of compounds 1-5 for their activity against *Shigella Castellani* 29
Figure S48. The inhibitory effect of compounds 4 and 5 on tumor cells KTC-1 and Hela 29

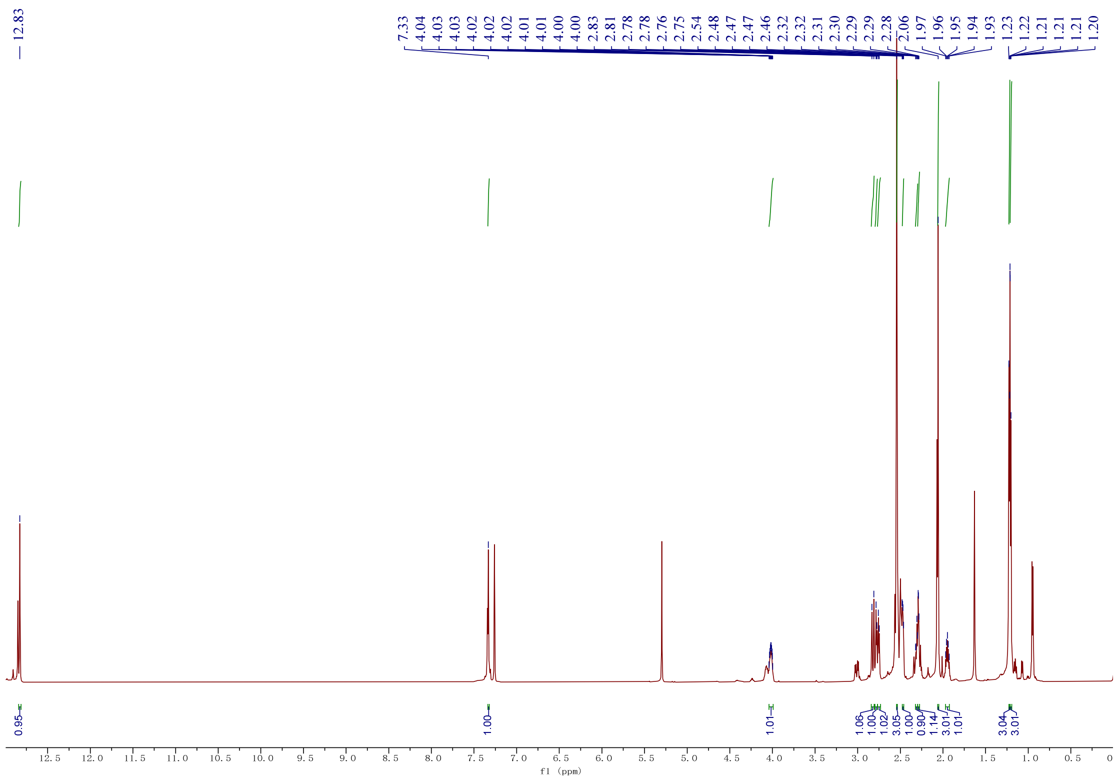

Figure S1. ^{1}H NMR spectrum (600MHz, $CDCl_3$) of **1**

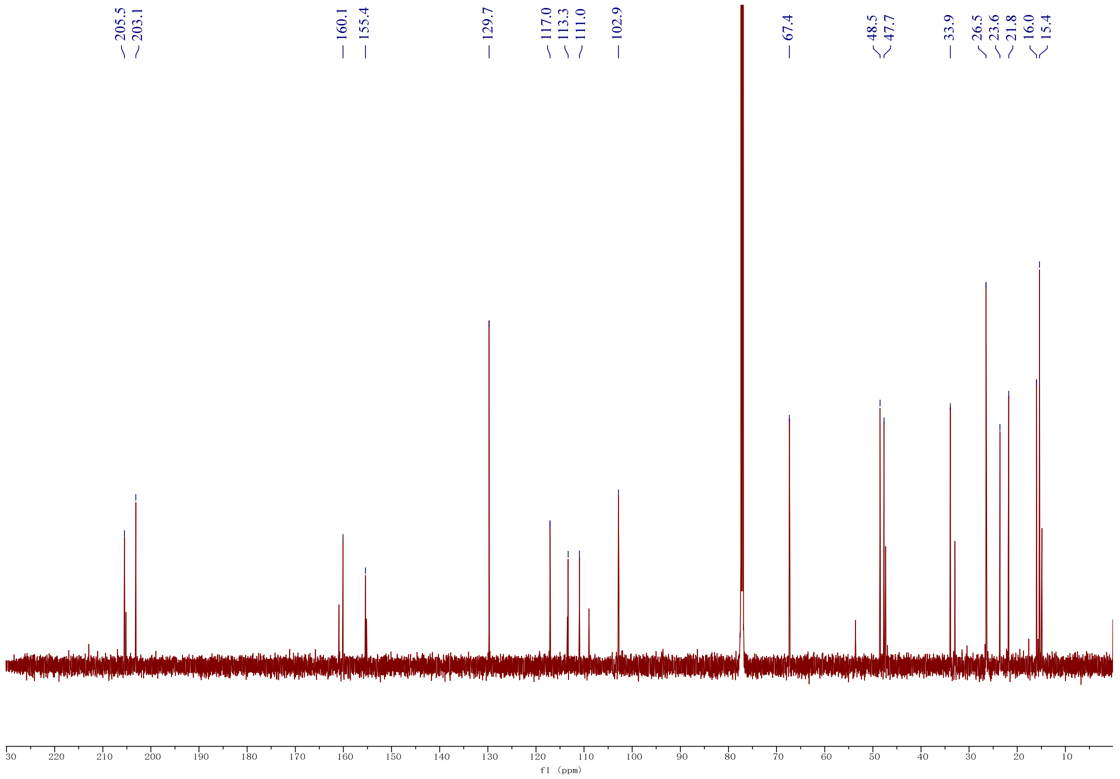

Figure S2. ^{13}C spectrum (150MHz, $CDCl_3$) of **1**

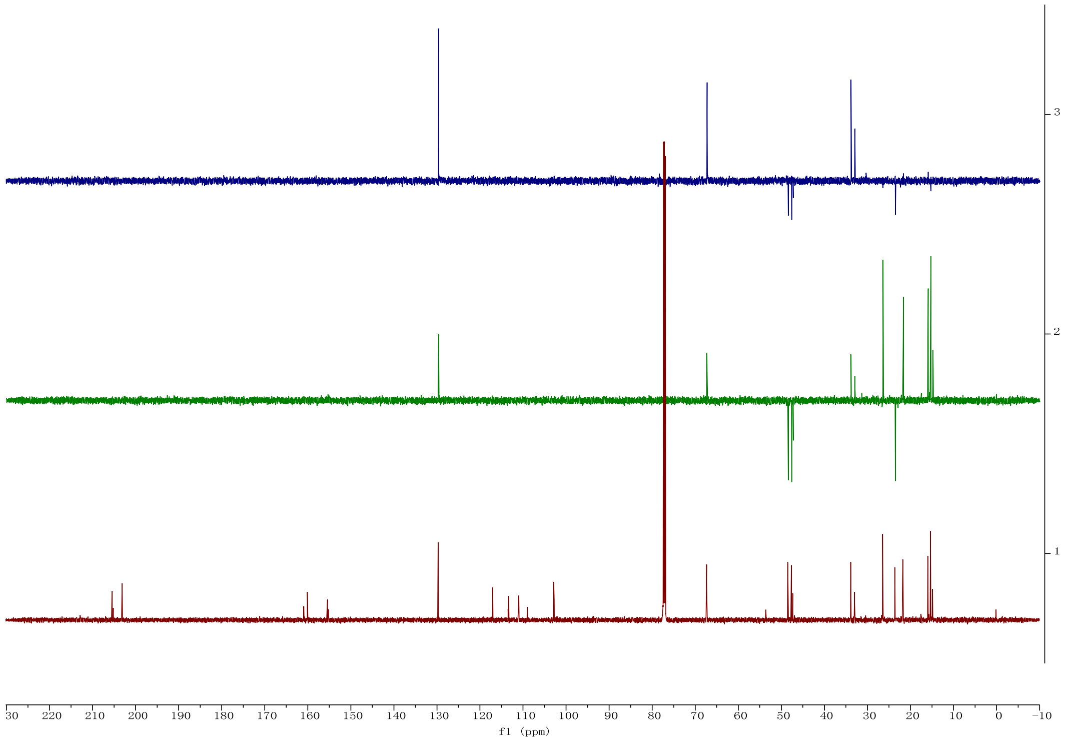

Figure S3. DEPT spectrum of **1**

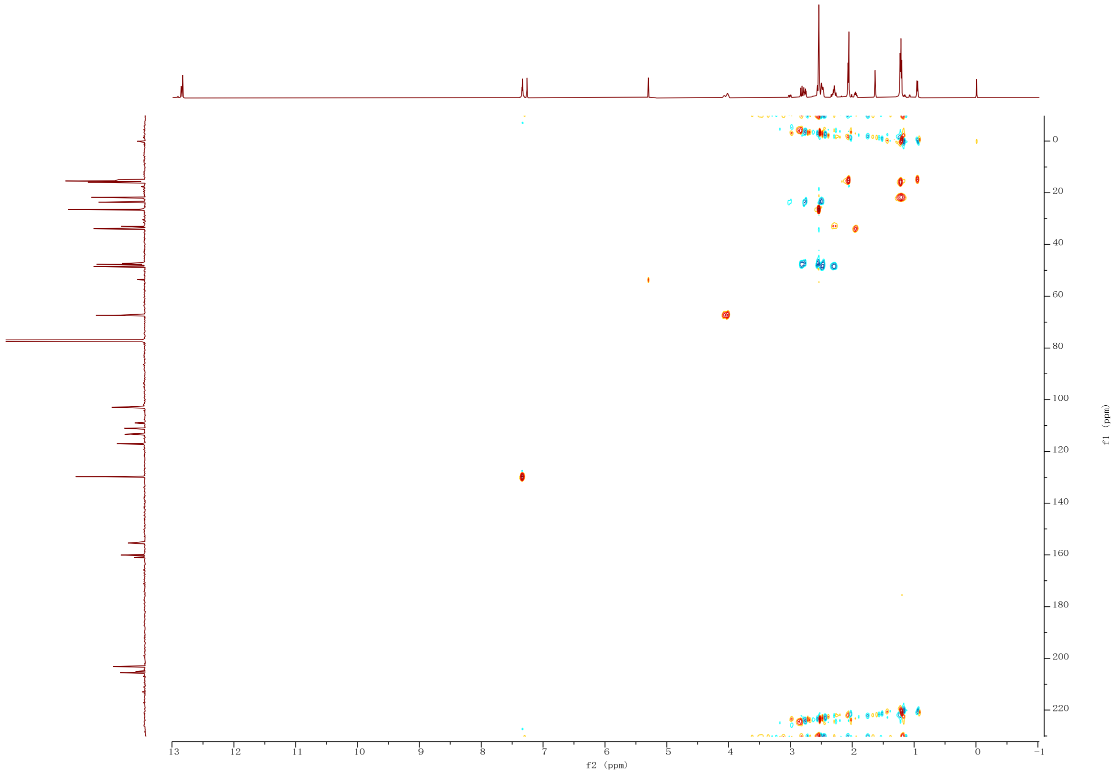

Figure S4. HSQC spectrum of **1**

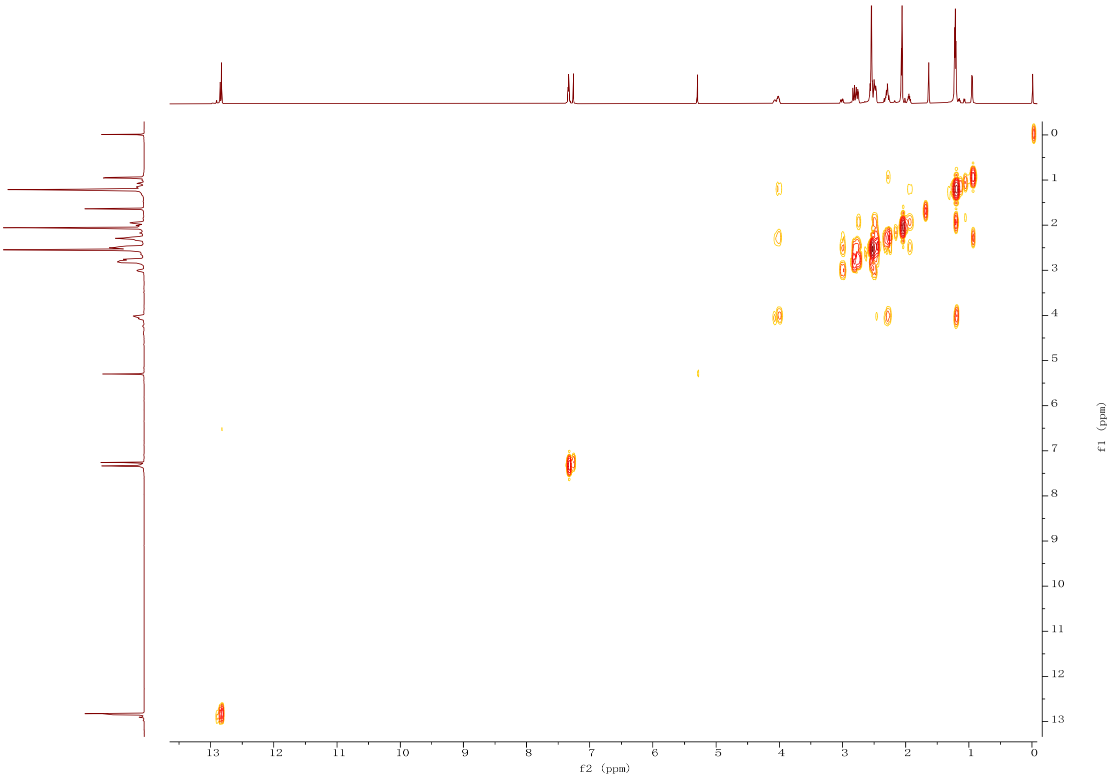

Figure S5. ^{1}H-^{1}H COSY spectrum of **1**

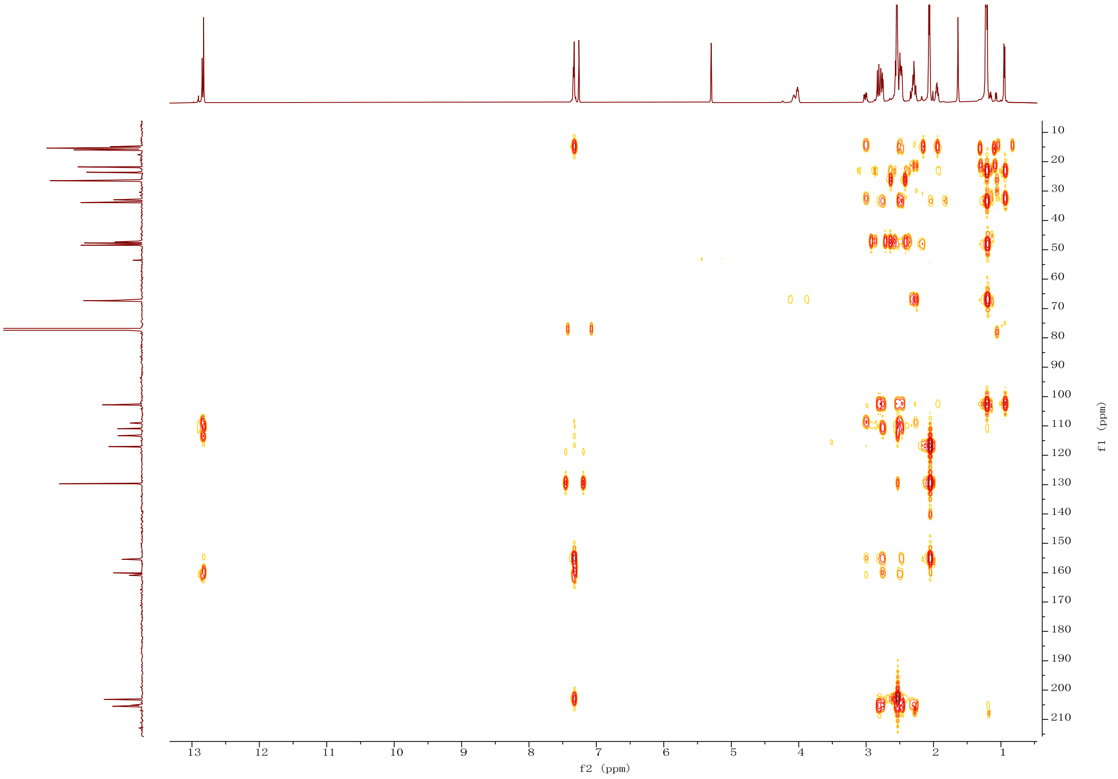

Figure S6. HMBC spectrum of **1**

Data File: E:\DATA\2023\0725\2\PC-44.lcd

Elmt	Val.	Min	Max	Elmt	Val.	Min	Max	Elmt	Val.	Min	Max	Elmt	Val.	Min	Max	Use Adduct
H	1	5	100	F	1	0	0	Cl	1	0	0	Ag	1	0	0	H
2H	1	0	0	Na	1	0	0	Co	2	0	0	I	3	0	5	Na
B	3	0	0	Mg	2	0	0	Cu	2	0	0	Ir	3	0	0	
C	4	5	100	Si	4	0	0	Se	2	0	0					
N	3	0	10	P	3	0	0	Br	1	0	5					
O	2	0	30	S	2	0	0	Pd	2	0	0					

Error Margin (ppm): 5
HC Ratio: unlimited
Max Isotopes: all
MSn Iso RI (%): 75.00

DBE Range: not fixed
Apply N Rule: yes
Isotope RI (%): 1.00
MSn Logic Mode: OR

Electron Ions: both
Use MSn Info: yes
Isotope Res: 10000
Max Results: 30

Event#: 1 MS(E+) Ret. Time : 0.400 -> 0.480 Scan# : 61 -> 73

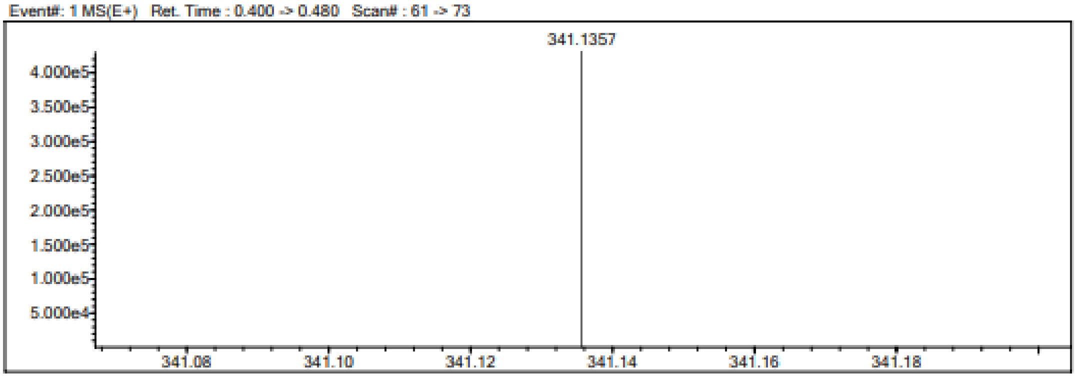

Measured region for 341.1357 m/z

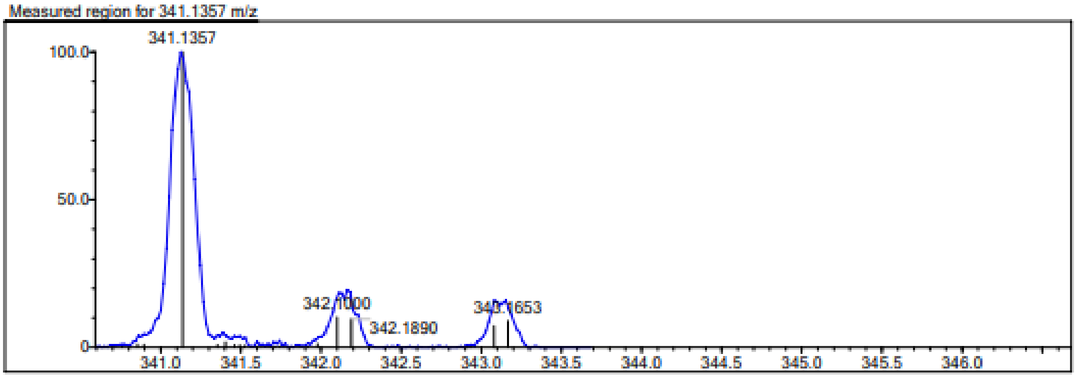

C18 H22 O5 [M+Na]+ : Predicted region for 341.1359 m/z

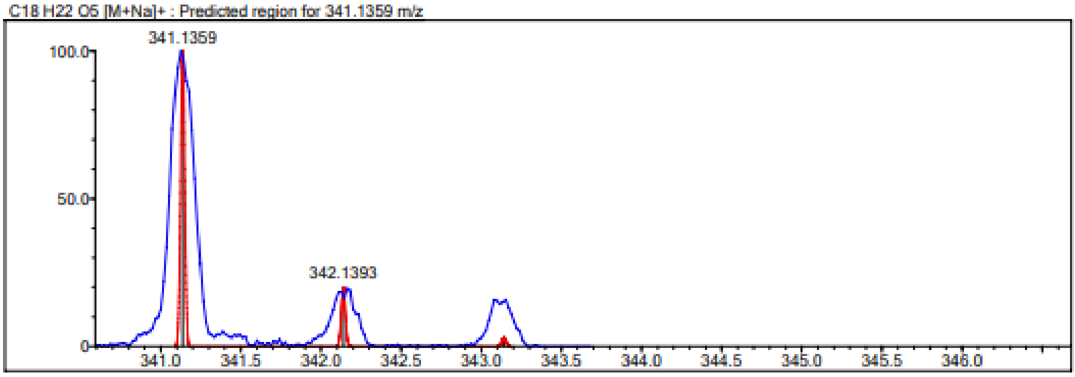

Formula (M)	Ion	Meas. m/z	Pred. m/z	Df. (mDa)	Df. (ppm)	DBE
C18 H22 O5	[M+Na]+	341.1357	341.1359	-0.2	-0.59	8.0

Figure S7. HR-ESI-MS spectrum of compound **1**

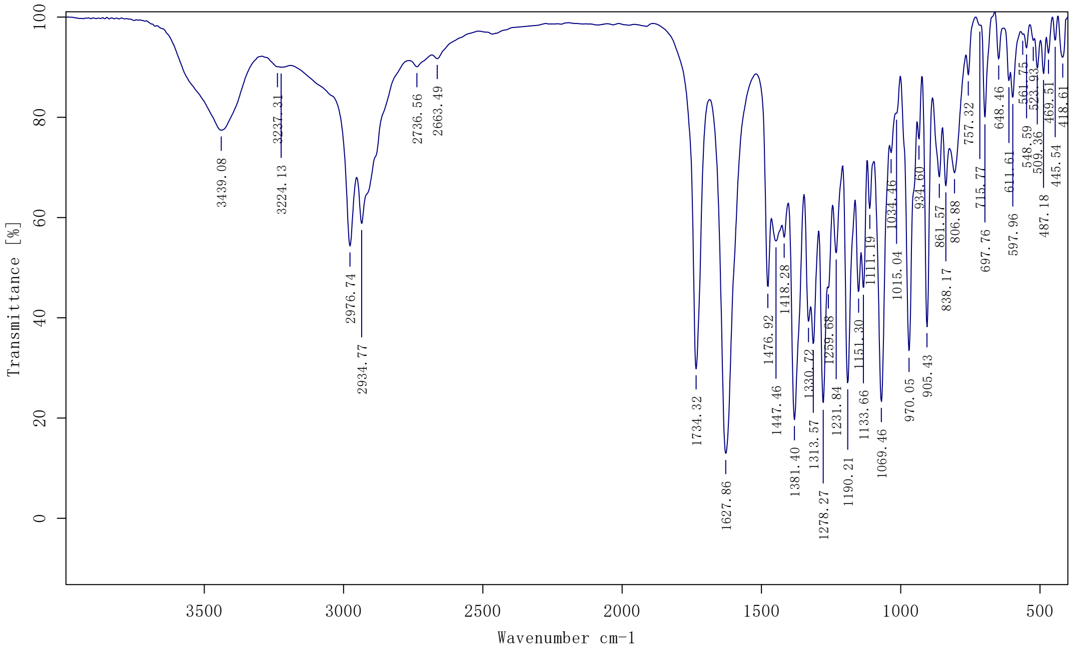

Sample Name: PC-44
Sample Form: KBr
Path of File: E:\data
Date of Measurement: 2023/7/30

Resolution: 4
Aperture Setting: 6 mm
Number of Background Scans: 16
Number of Sample Scans: 16

Beamsplitter Setting: KBr
Source Setting: MIR
Instrument Type: BRUKER VERTEX 70
Soft Version: OPUS8.1

Figure S8. IR spectrum of compound **1**

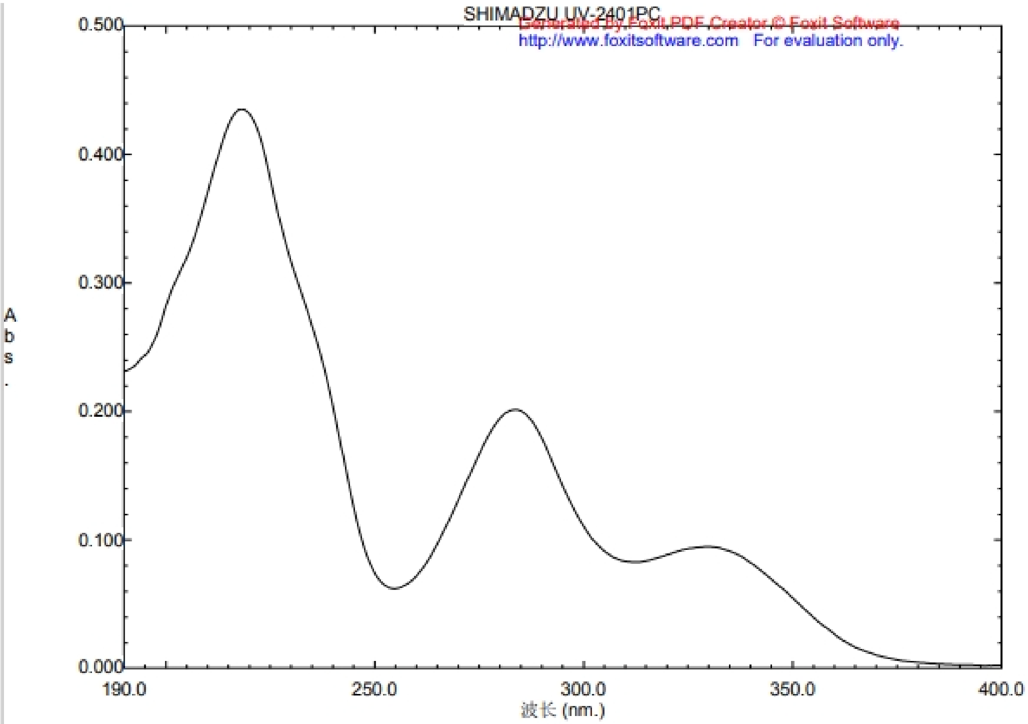

Figure S9. UV spectrum of compound **1**

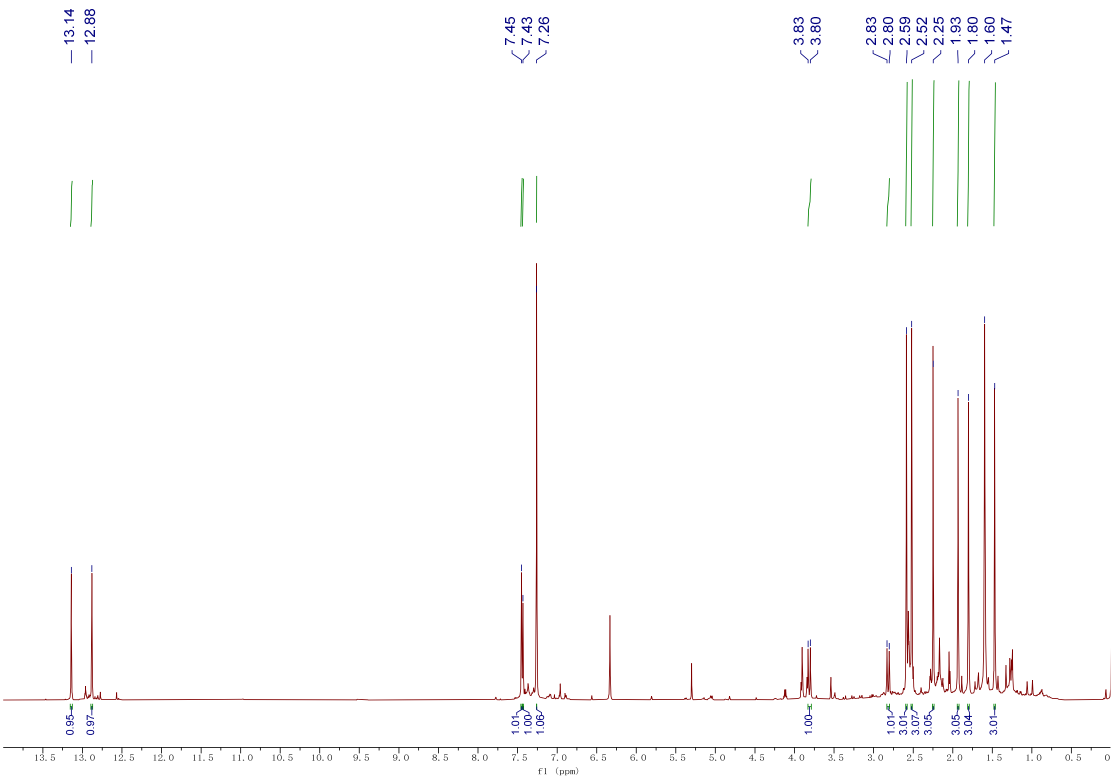

Figure S10. ^{1}H NMR spectrum (600MHz, $CDCl_3$) of **2**

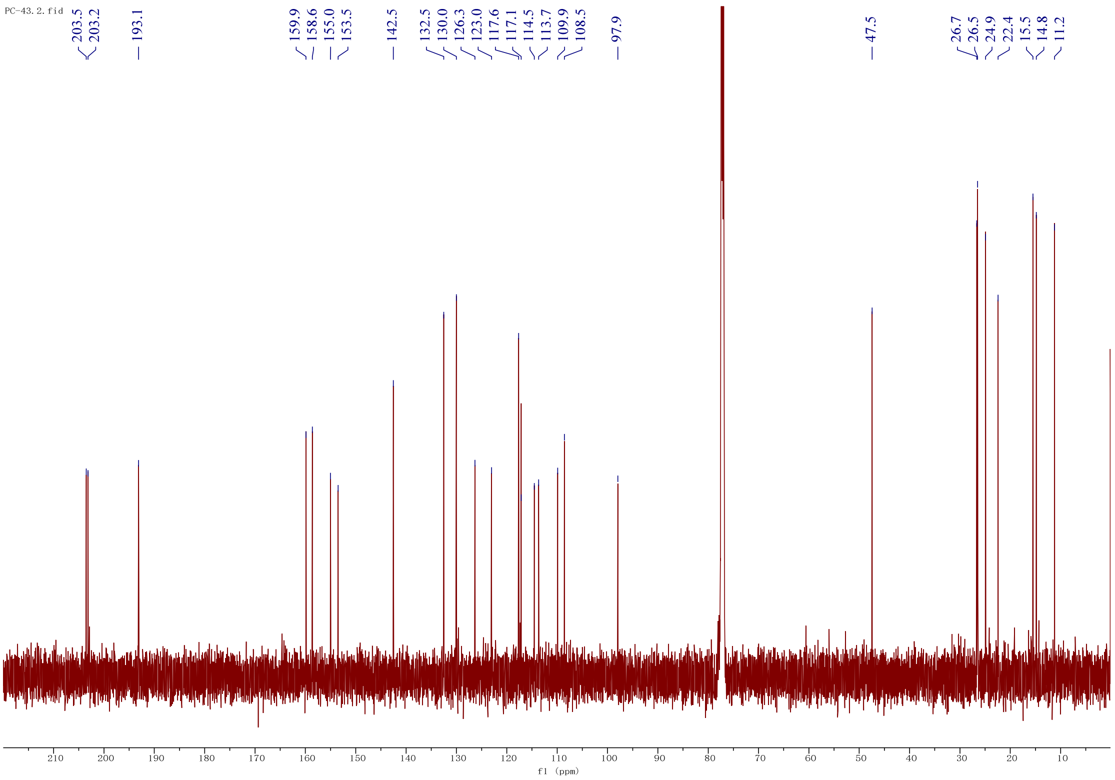

Figure S11. ^{13}C spectrum (150MHz, $CDCl_3$) of **2**

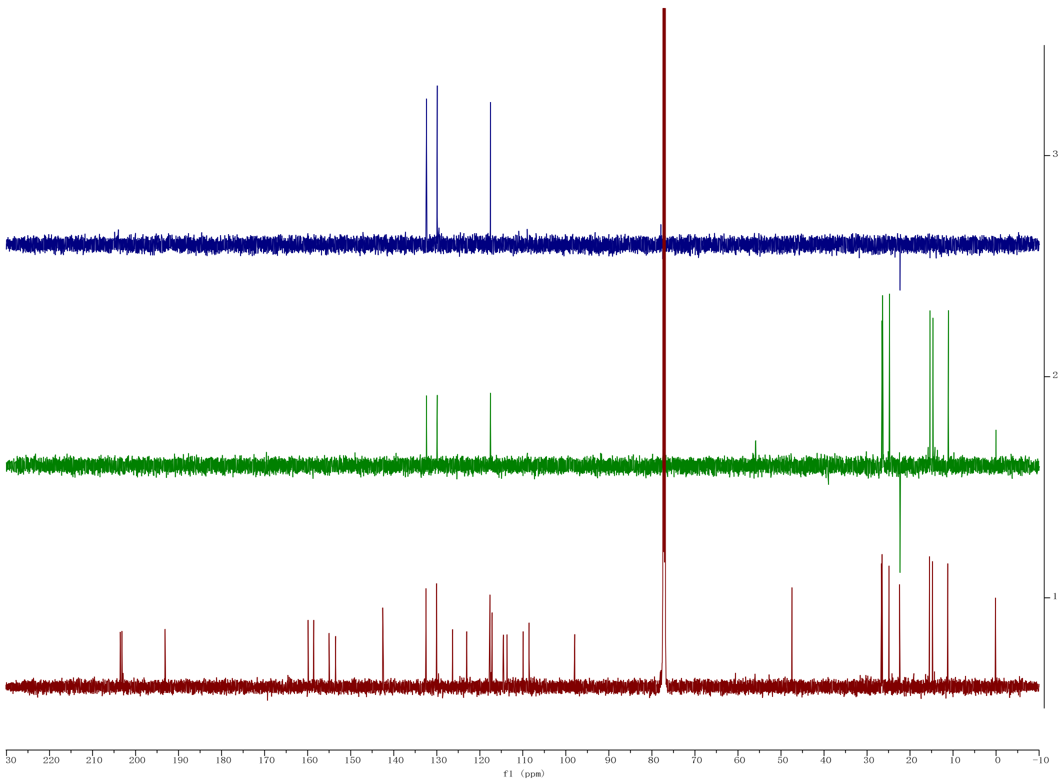

Figure S12. DEPT spectrum (150MHz, $CDCl_3$) of **2**

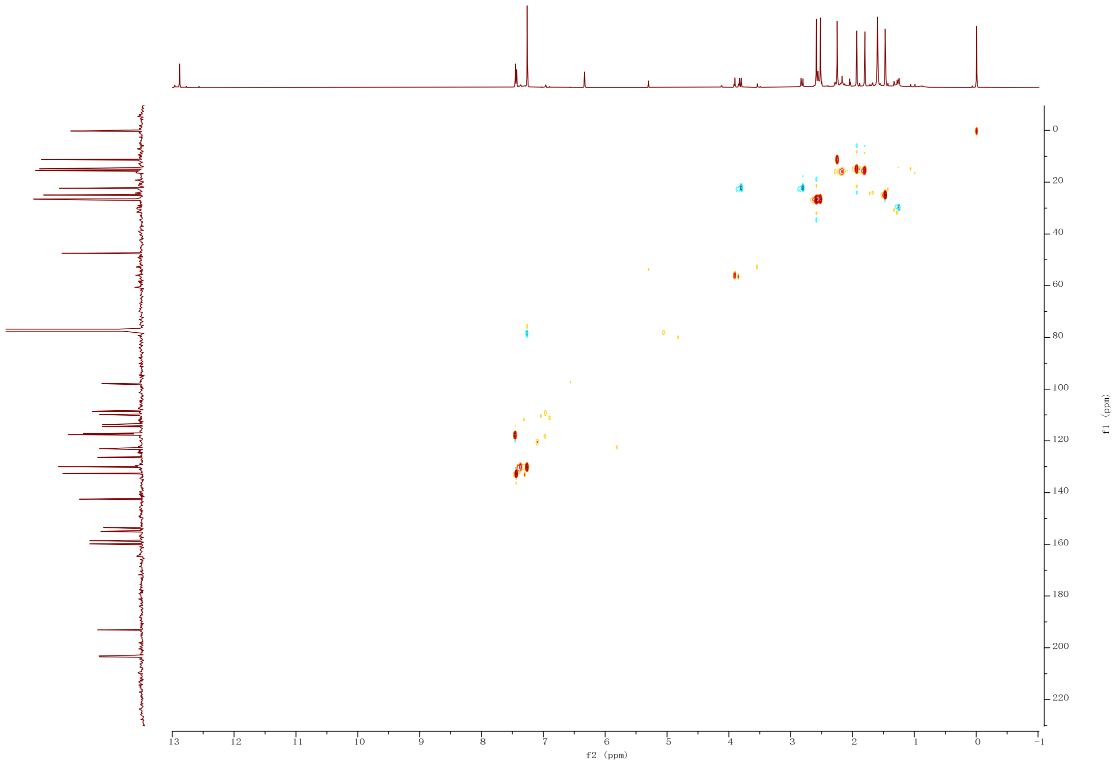

Figure S13. HSQC spectrum of **2**

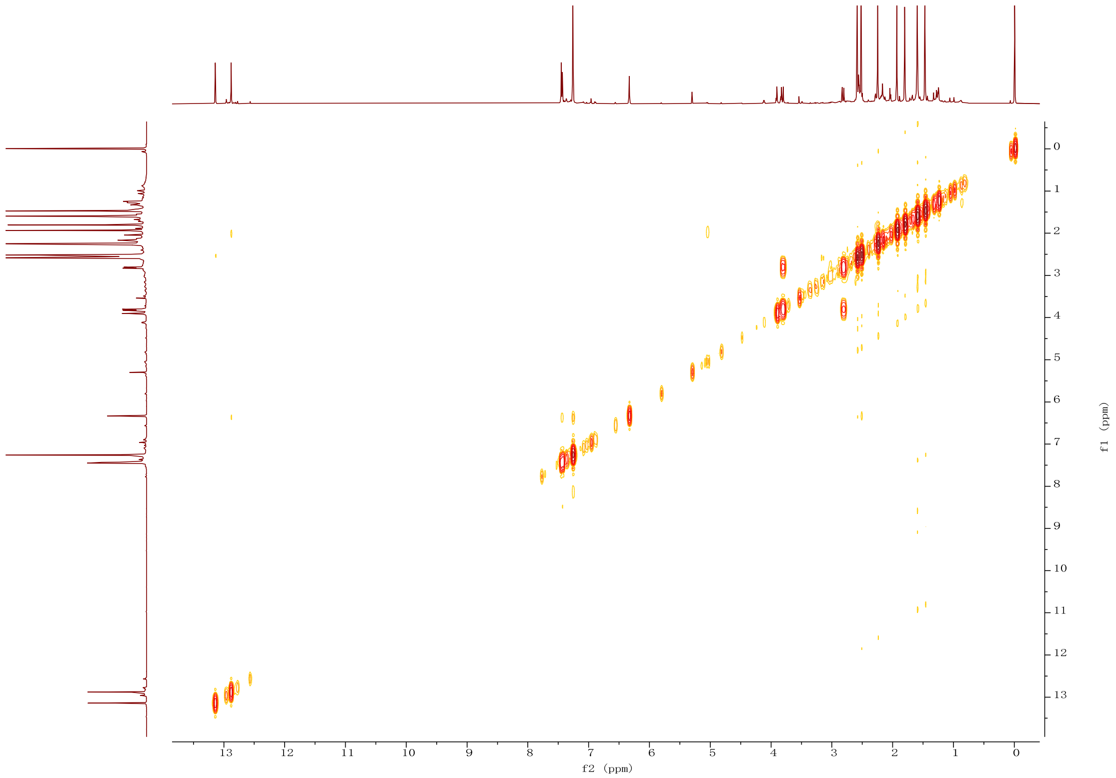

Figure S14. ^{1}H-^{1}H COSY spectrum of **2**

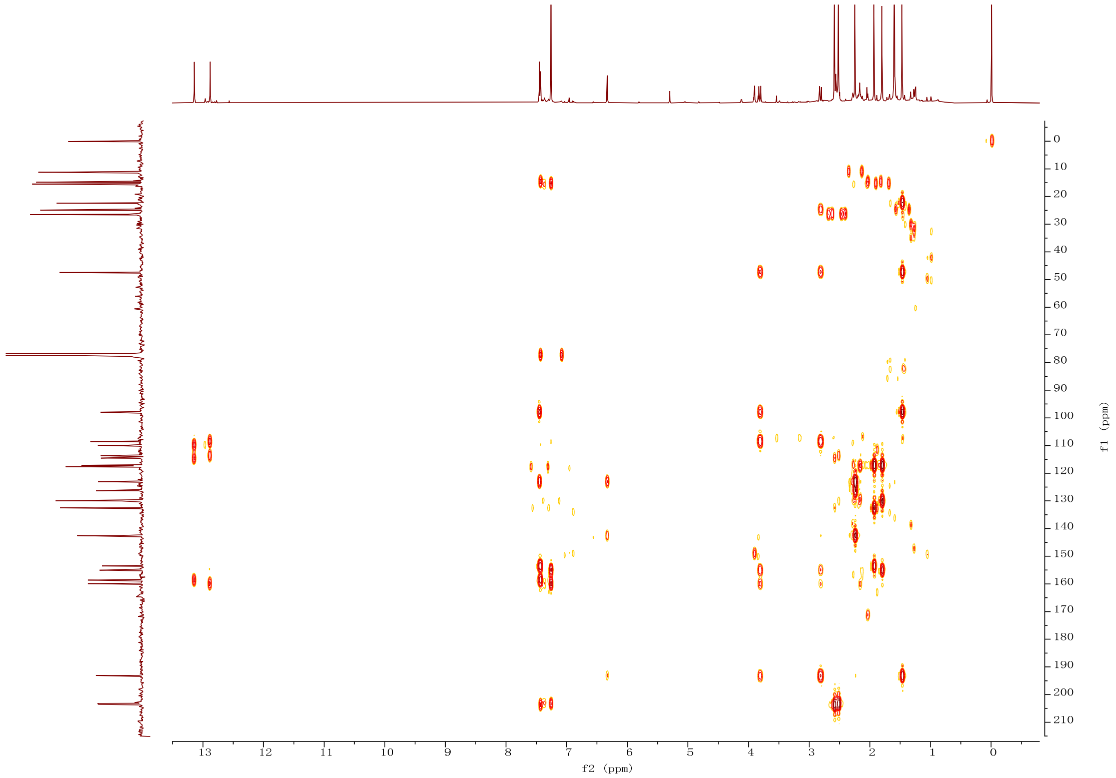

Figure S15. HMBC spectrum of **2**

Data File: E:\DATA\2023\0911\1\PC-43.lcd

Elmt	Val.	Min	Max	Elmt	Val.	Min	Max	Elmt	Val.	Min	Max	Elmt	Val.	Min	Max	Use Adduct
H	1	5	100	F	1	0	0	Cl	1	0	0	Ag	1	0	0	H
2H	1	0	0	Na	1	0	0	Co	2	0	0	I	3	0	0	Na
B	3	0	0	Mg	2	0	0	Cu	2	0	0	Ir	3	0	0	
C	4	5	100	Si	4	0	0	Se	2	0	0					
N	3	0	10	P	3	0	0	Br	1	0	0					
O	2	0	30	S	2	0	0	Pd	2	0	0					

Error Margin (ppm): 5
HC Ratio: unlimited
Max Isotopes: all
MSn Iso RI (%): 75.00

DBE Range: not fixed
Apply N Rule: yes
Isotope RI (%): 1.00
MSn Logic Mode: OR

Electron Ions: both
Use MSn Info: yes
Isotope Res: 10000
Max Results: 30

Event#: 1 MS(E+) Ret. Time : 0.347 -> 0.613 Scan# : 53 -> 93

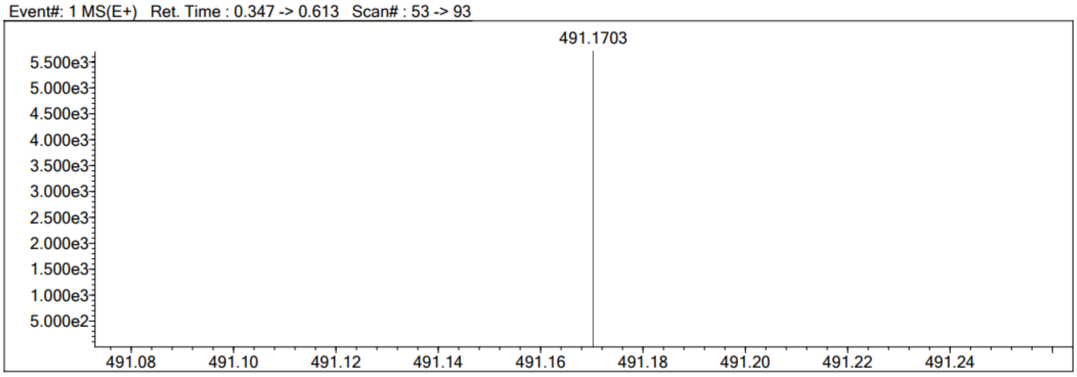

Measured region for 491.1703 m/z

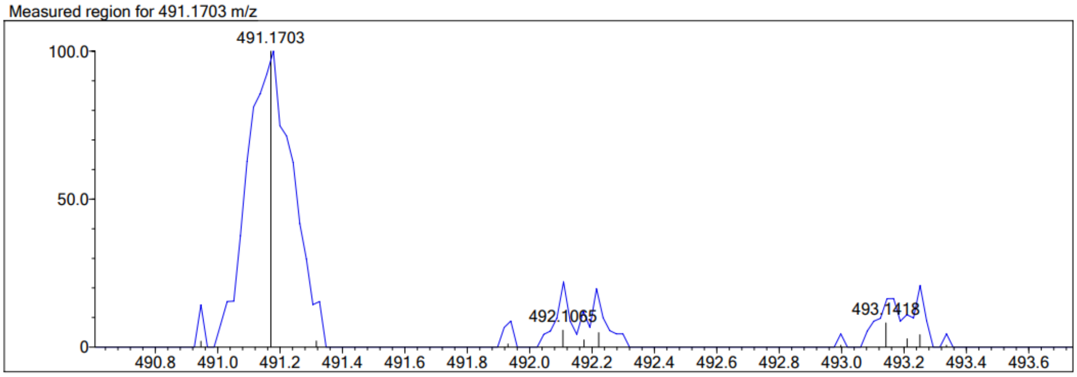

C28 H26 O8 [M+H]+ : Predicted region for 491.1700 m/z

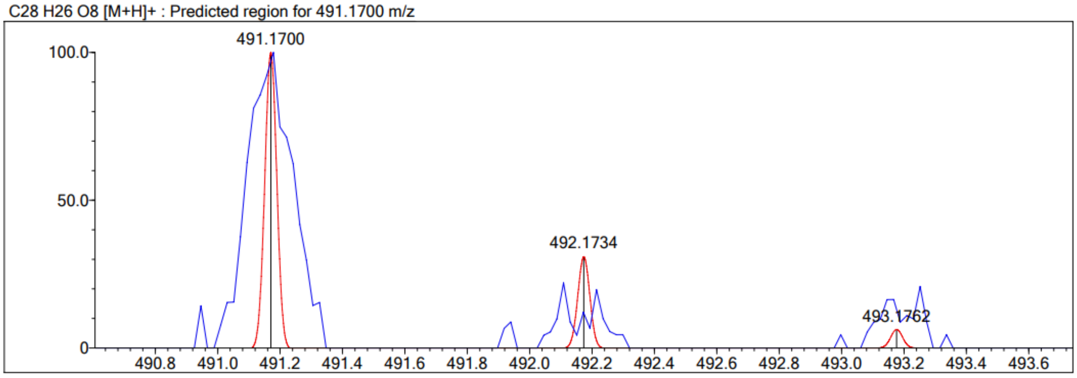

Formula (M)	Ion	Meas. m/z	Pred. m/z	Df. (mDa)	Df. (ppm)	DBE
C28 H26 O8	[M+H]+	491.1703	491.1700	0.3	0.61	16.0

Figure S16. HR-ESI-MS spectrum of compound **2**

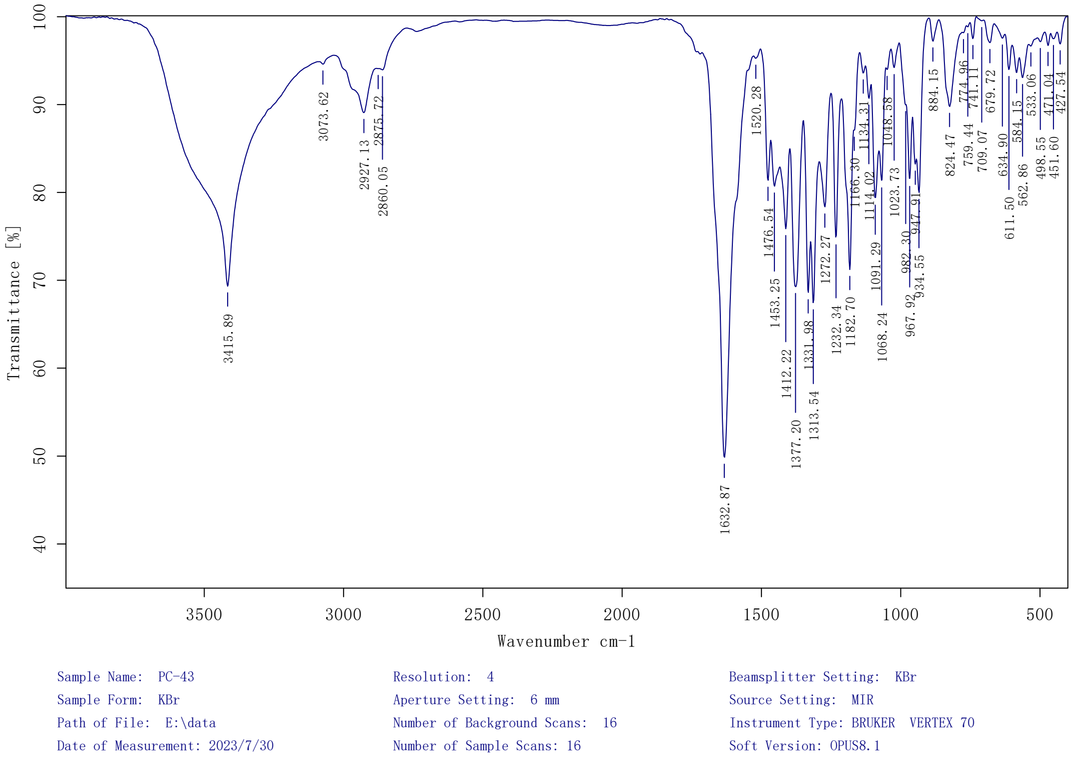

Figure S17. IR spectrum of compound **2**

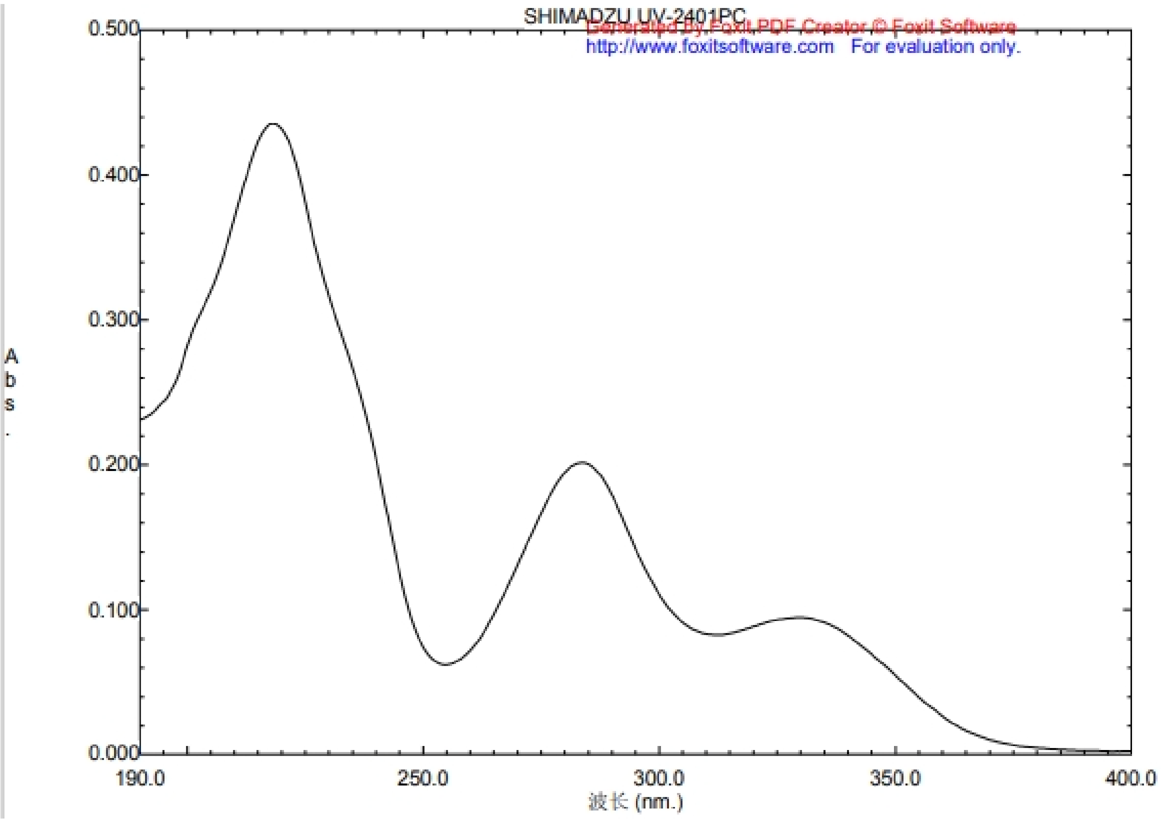

Figure S18. UV spectrum of compound **2**

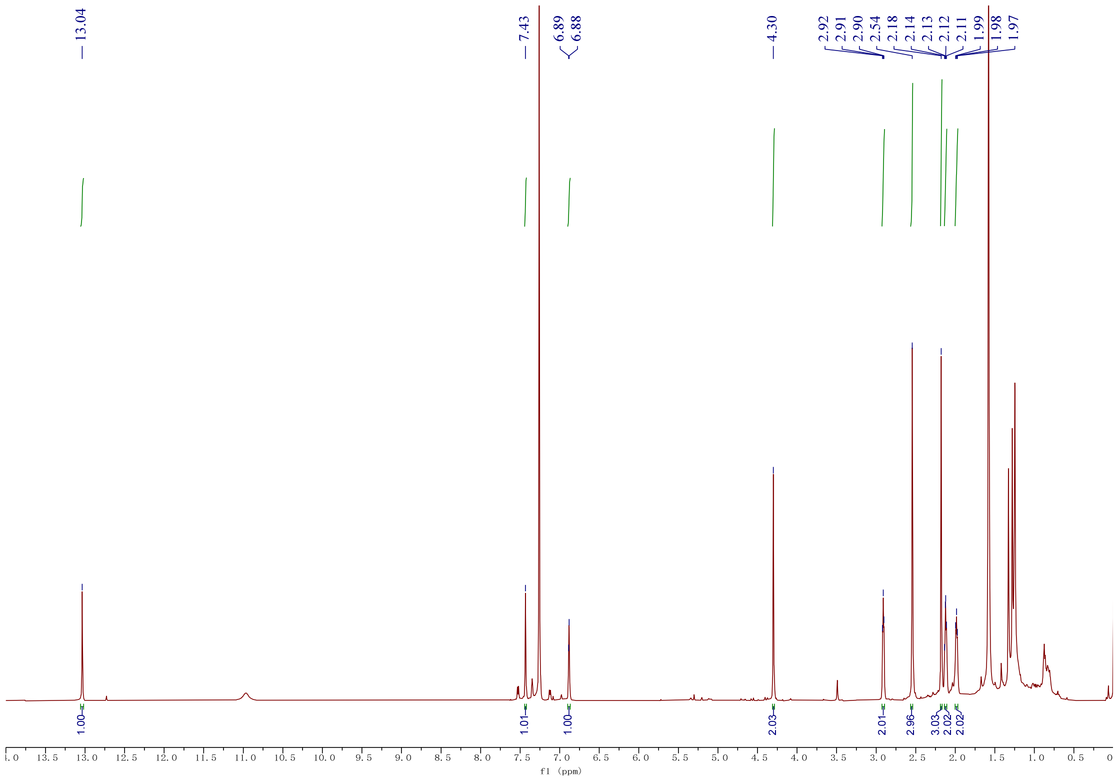

Figure S19. ^{1}H NMR spectrum (600MHz, $CDCl_3$) of **3**

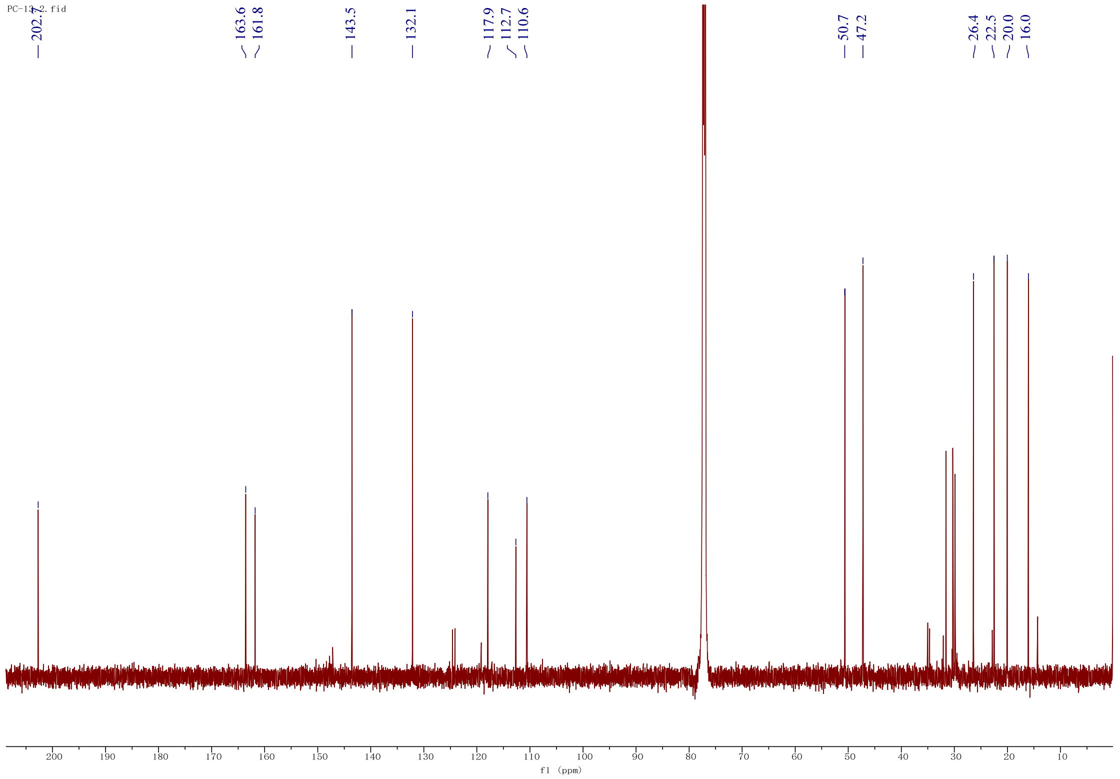

Figure S20. ^{13}C spectrum (150MHz, $CDCl_3$) of **3**

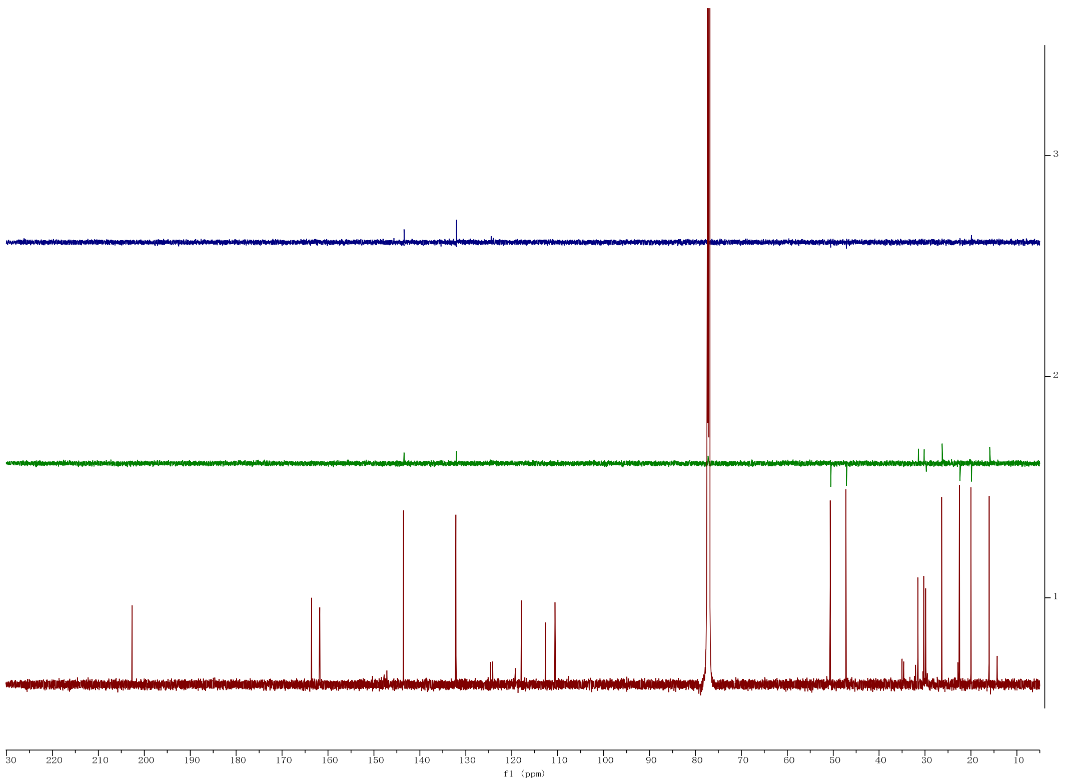

Figure S21. DEPT spectrum (150MHz, $CDCl_3$) of **3**

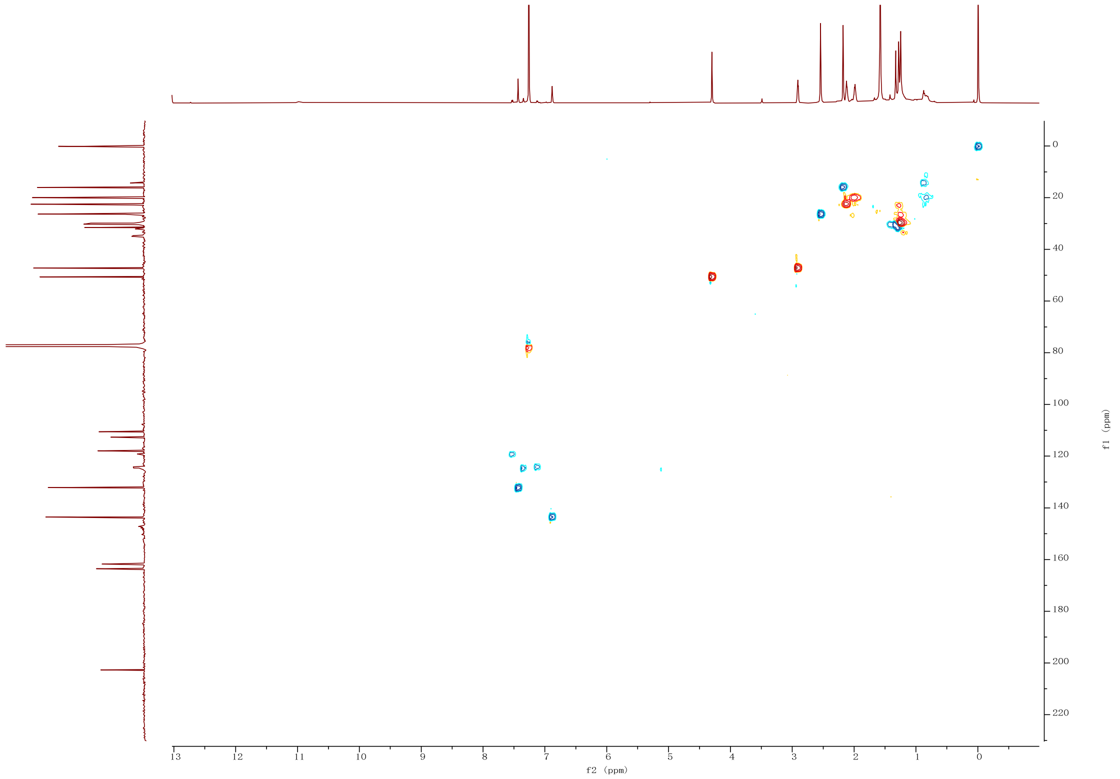

Figure S22. HSQC spectrum of **3**

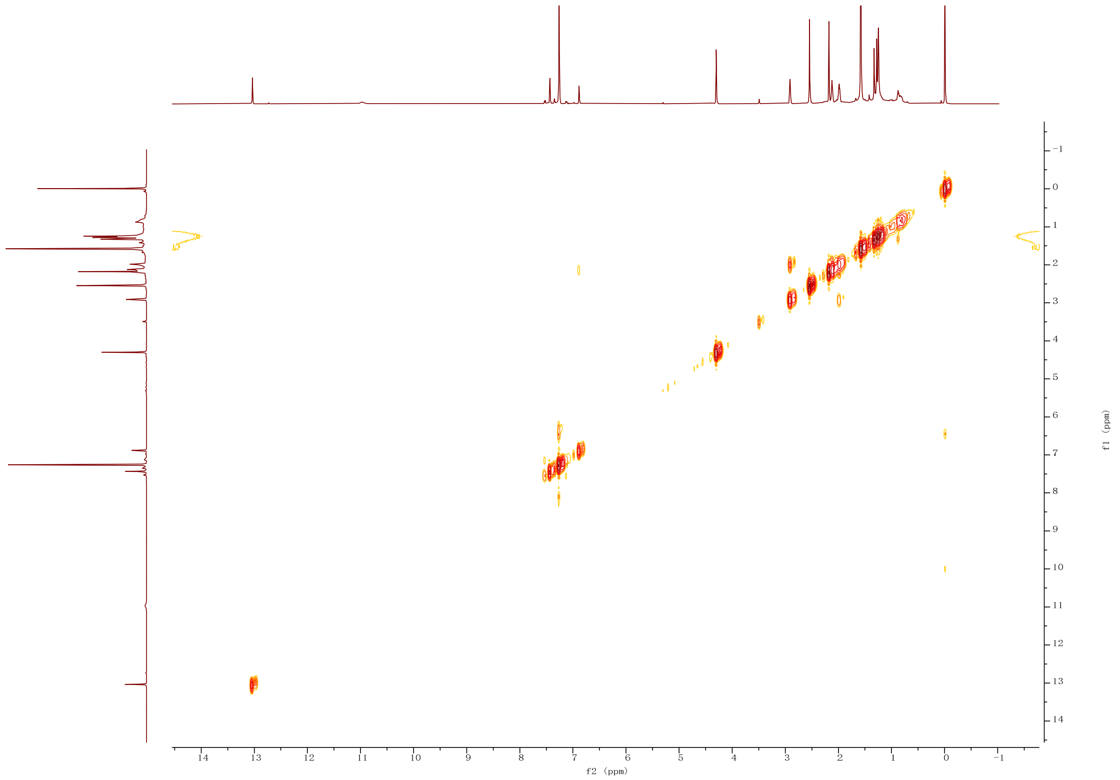

Figure S23. ^{1}H-^{1}H COSY spectrum of **3**

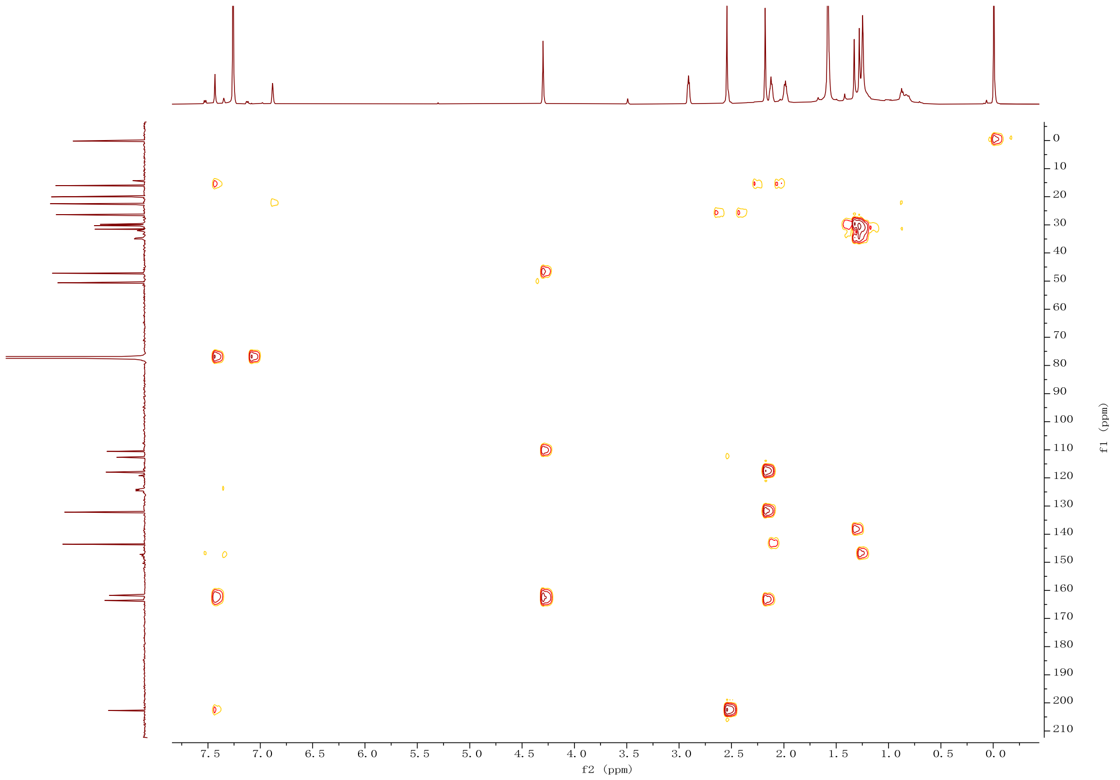

Figure S24. HMBC spectrum of **3**

Data File: E:\DATA\2023\0703\PC-13.lcd

Elmt	Val.	Min	Max	Elmt	Val.	Min	Max	Elmt	Val.	Min	Max	Elmt	Val.	Min	Max	Use Adduct
H	1	5	100	F	1	0	5	Cl	1	0	0	Ag	1	0	0	H
2H	1	0	0	Na	1	0	0	Co	2	0	0	I	3	0	5	
B	3	0	0	Mg	2	0	0	Cu	2	0	0	Ir	3	0	0	
C	4	5	100	Si	4	0	0	Se	2	0	0					
N	3	0	10	P	3	0	0	Br	1	0	0					
O	2	0	30	S	2	0	5	Pd	2	0	0					

Error Margin (ppm):	5	DBE Range:	not fixed	Electron Ions:	both
HC Ratio:	unlimited	Apply N Rule:	no	Use MSn Info:	yes
Max Isotopes:	all	Isotope RI (%):	1.00	Isotope Res:	10000
MSn Iso RI (%):	75.00	MSn Logic Mode:	OR	Max Results:	30

Event#: 2 MS(E-) Ret. Time : 0.373 -> 0.400 Scan# : 58 -> 62

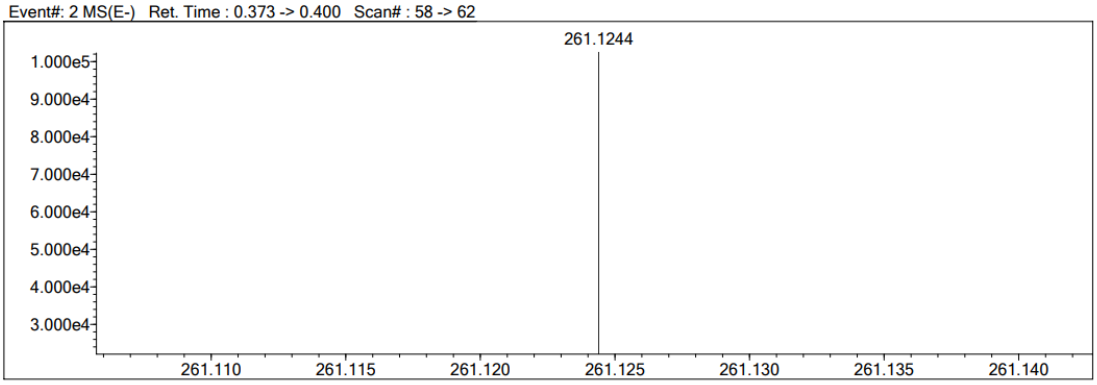

Measured region for 261.1244 m/z

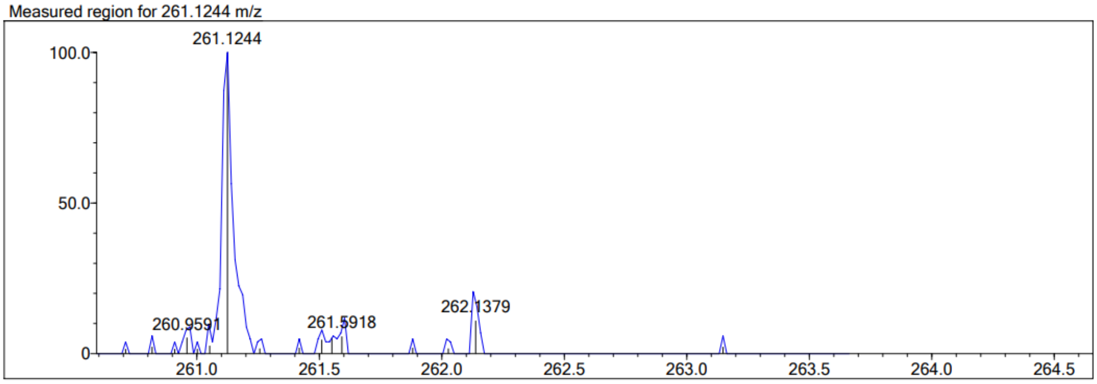

C14 H18 N2 O3 [M-H]- : Predicted region for 261.1245 m/z

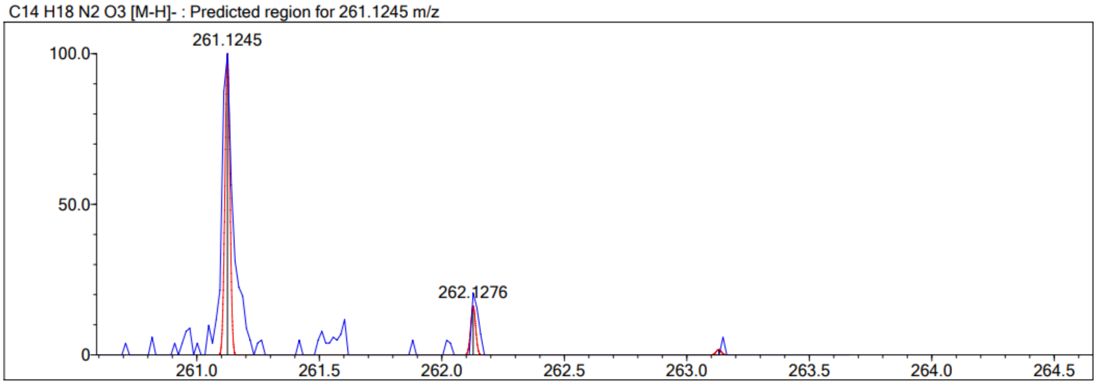

Formula (M)	Ion	Meas. m/z	Pred. m/z	Df. (mDa)	Df. (ppm)	DBE
C14 H18 N2 O3	[M-H]-	261.1244	261.1245	-0.1	-0.38	7.0

Figure S25. HR-ESI-MS spectrum of compound **3**

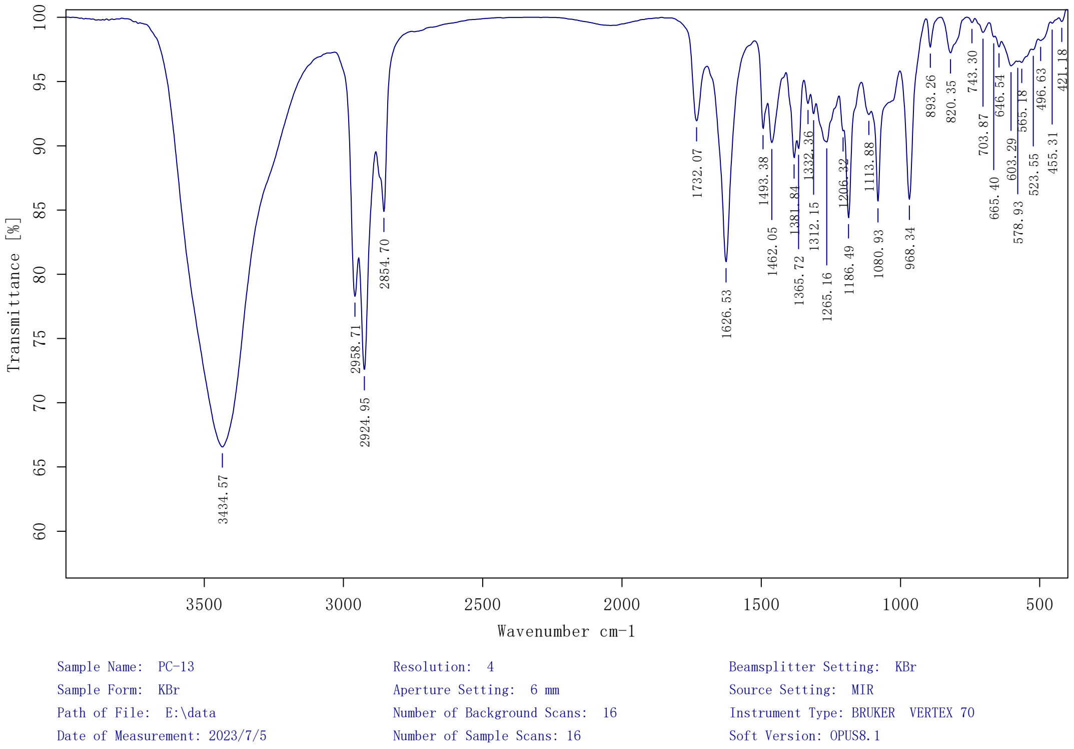

Figure S26. IR spectrum of compound **3**

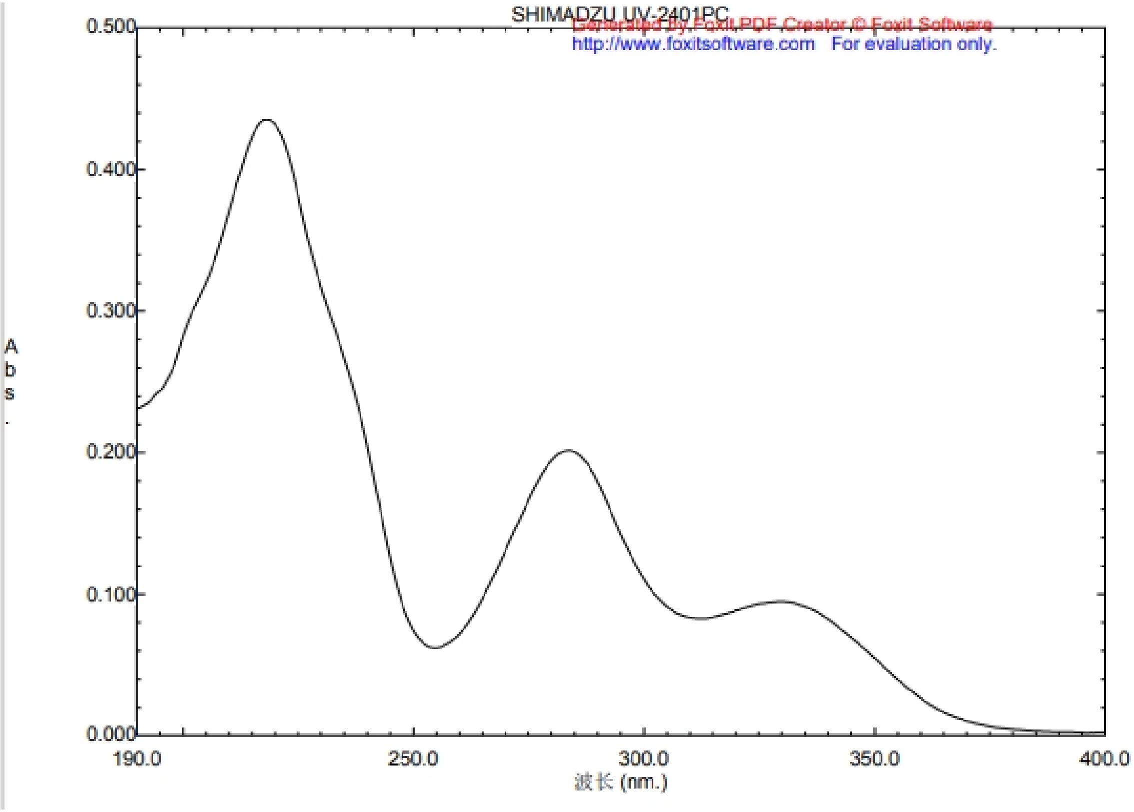

Figure S27. UV spectrum of compound **3**

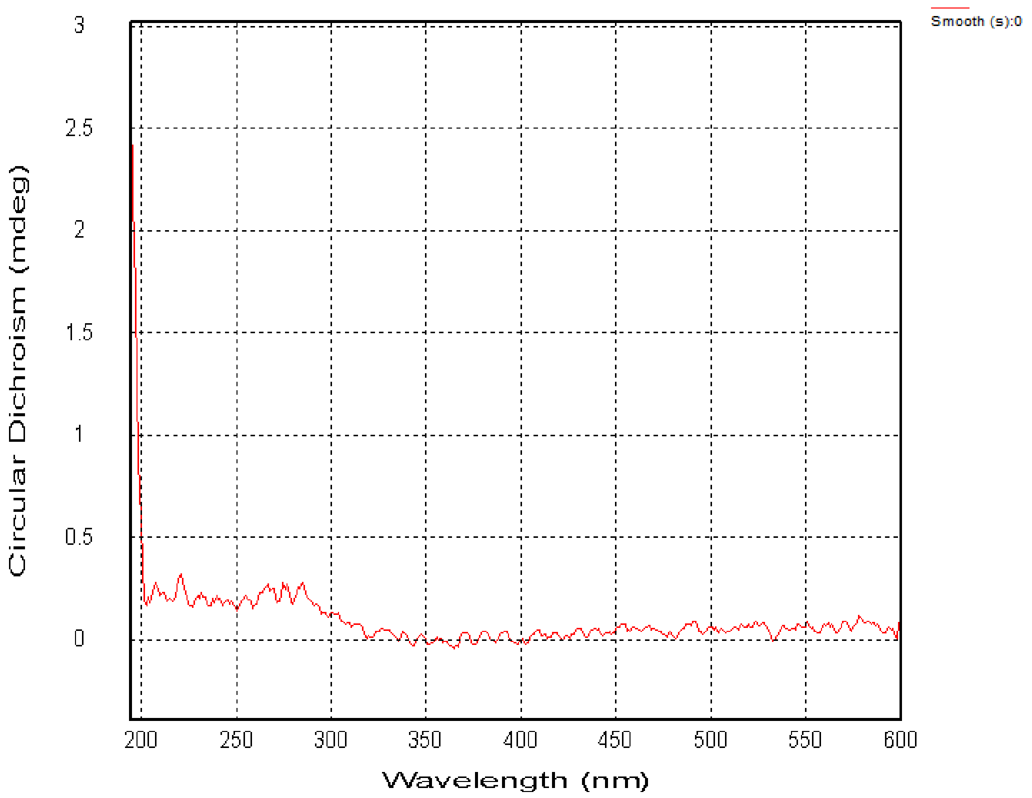

Figure S28. CD spectra of compound **3**

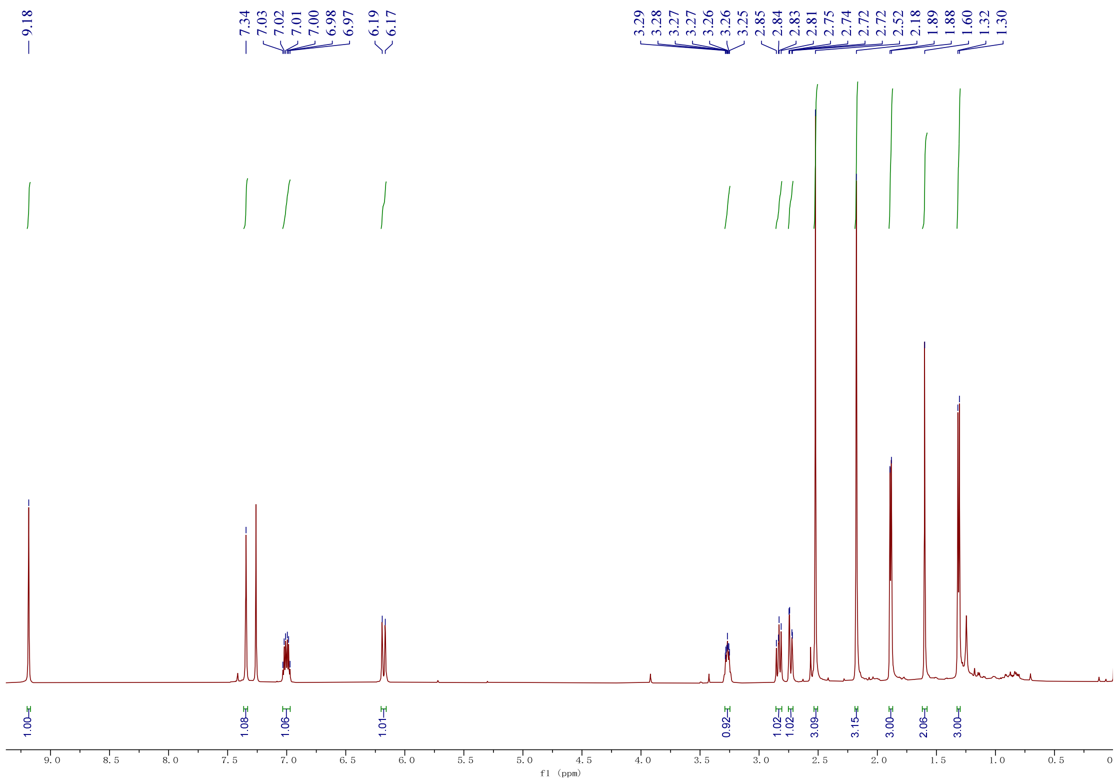

Figure S29. ^{1}H NMR spectrum (600MHz, $CDCl_3$) of **4**

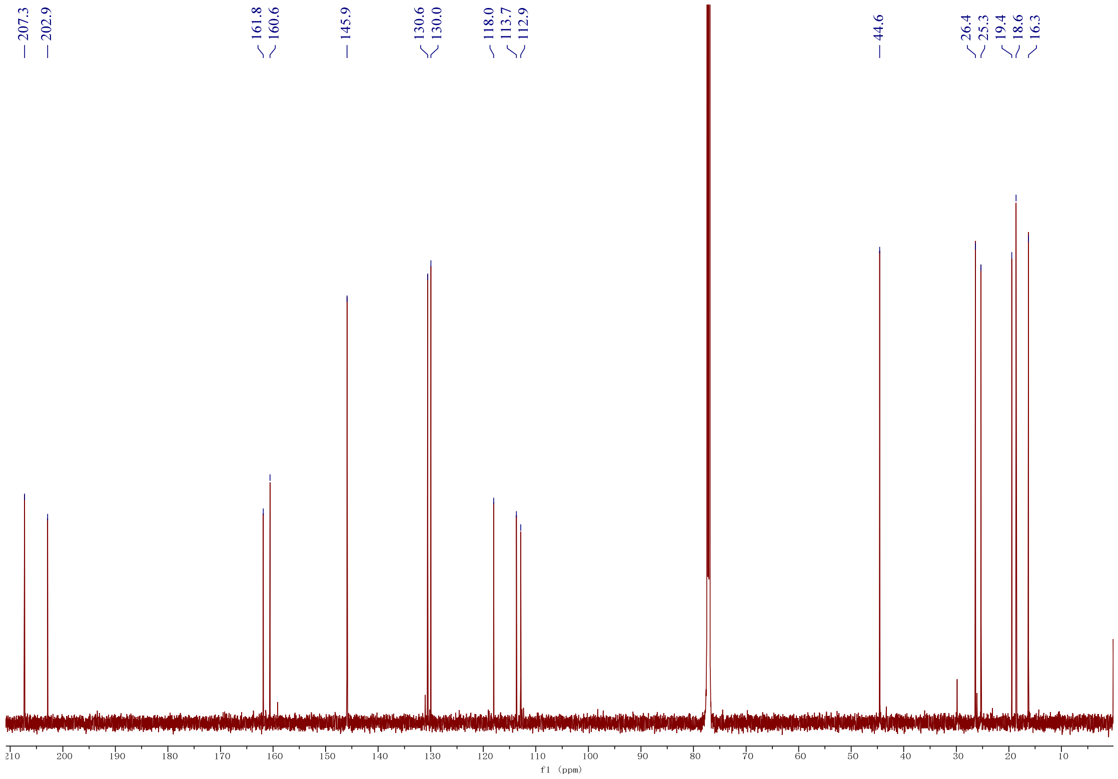

Figure S30. ^{13}C spectrum (150MHz, $CDCl_3$) of **4**

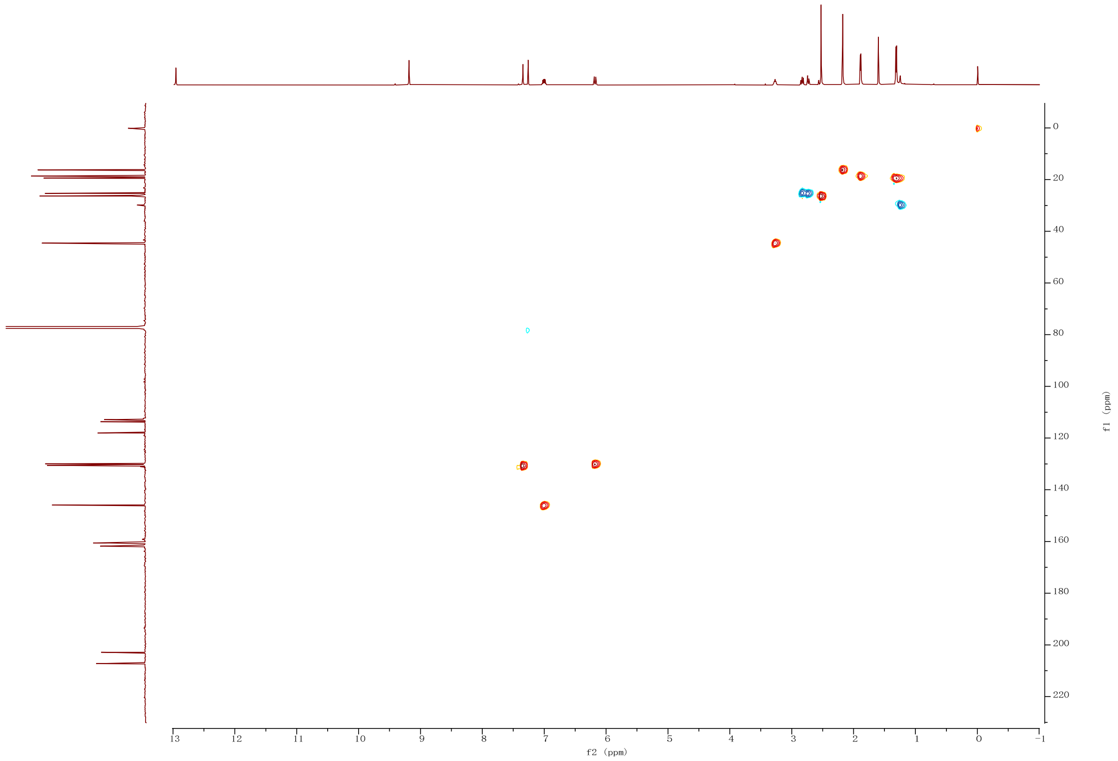

Figure S31. HSQC spectrum of **4**

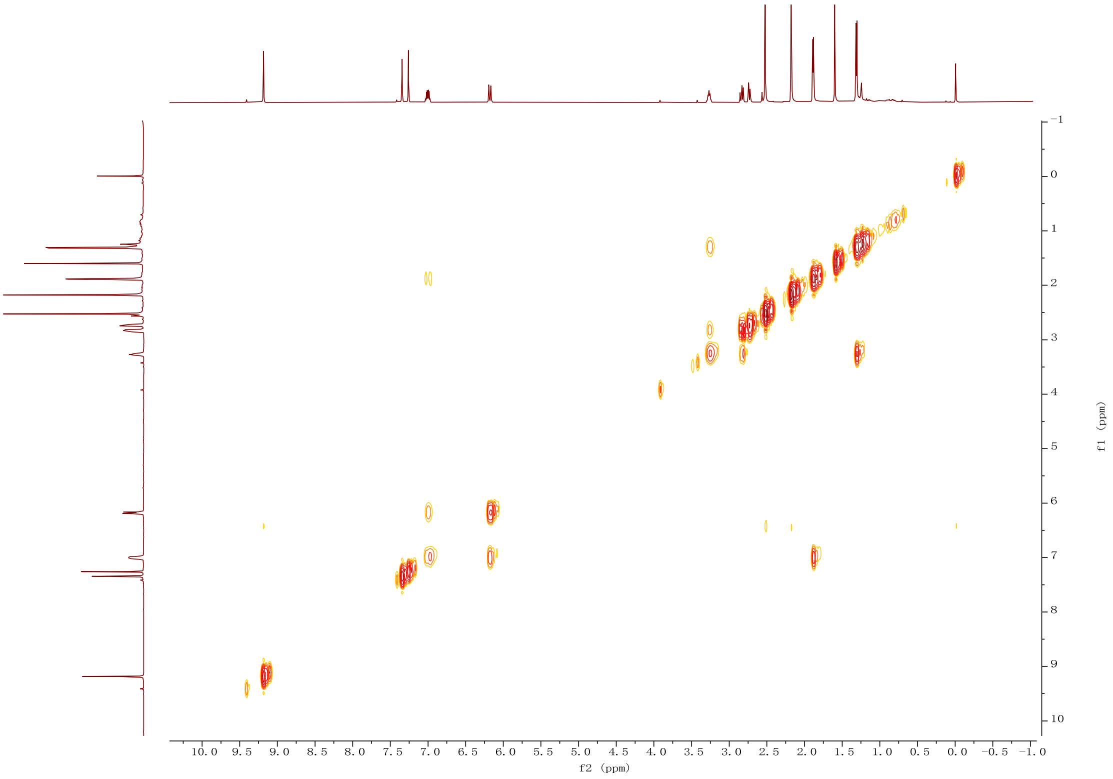

Figure S32. ^{1}H-^{1}H COSY spectrum of **4**

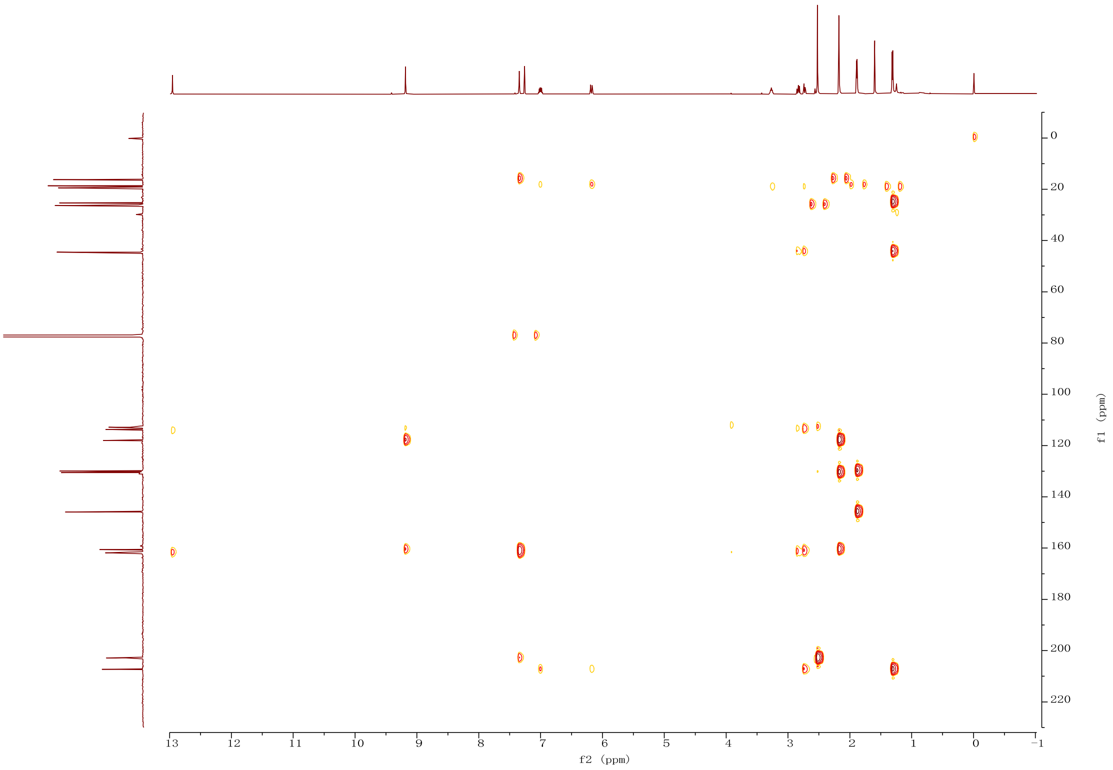

Figure S33. HMBC spectrum of **4**

Formula Predictor Report - PC-20.lcd Page 1 of 1

Data File: E:\DATA\2023\0703\PC-20.lcd

Elmt	Val.	Min	Max	Elmt	Val.	Min	Max	Elmt	Val.	Min	Max	Elmt	Val.	Min	Max	Use Adduct
H	1	5	100	F	1	0	5	Cl	1	0	0	Ag	1	0	0	H
2H	1	0	0	Na	1	0	0	Co	2	0	0	I	3	0	5	
B	3	0	0	Mg	2	0	0	Cu	2	0	0	Ir	3	0	0	
C	4	5	100	Si	4	0	0	Se	2	0	0					
N	3	0	10	P	3	0	0	Br	1	0	0					
O	2	0	30	S	2	0	5	Pd	2	0	0					

Error Margin (ppm):	5	DBE Range:	not fixed	Electron Ions:	both
HC Ratio:	unlimited	Apply N Rule:	no	Use MSn Info:	yes
Max Isotopes:	all	Isotope RI (%):	1.00	Isotope Res:	10000
MSn Iso RI (%):	75.00	MSn Logic Mode:	OR	Max Results:	30

Event#: 2 MS(E-) Ret. Time : 0.387 Scan# : 60

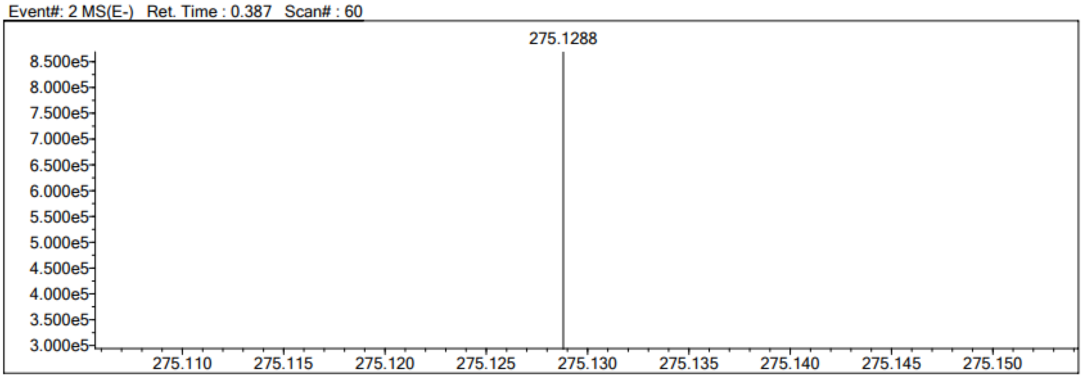

Measured region for 275.1288 m/z

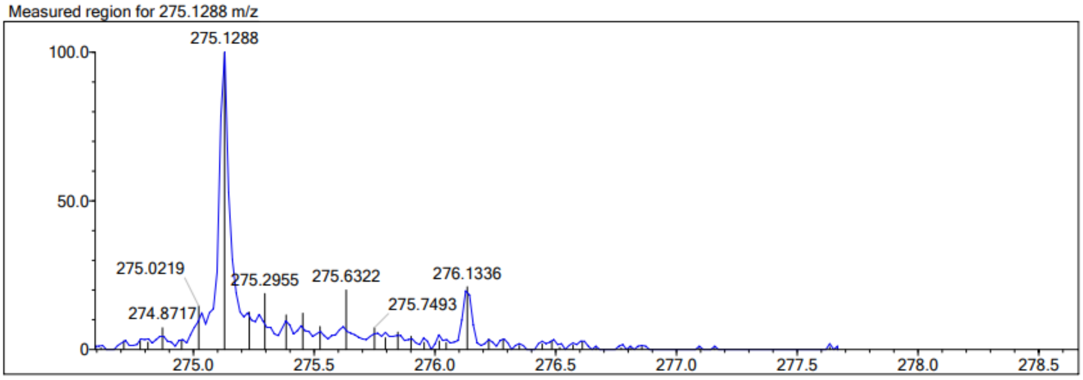

C16 H20 O4 [M-H]- : Predicted region for 275.1289 m/z

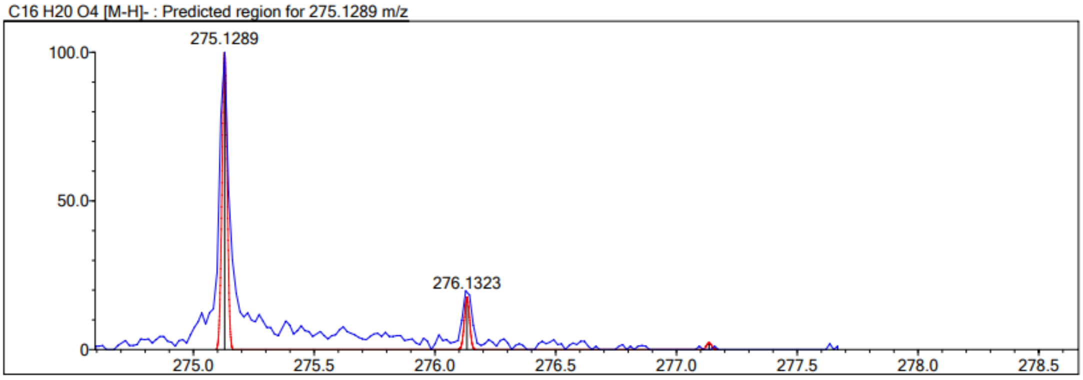

Formula (M)	Ion	Meas. m/z	Pred. m/z	Df. (mDa)	Df. (ppm)	DBE
C16 H20 O4	[M-H]-	275.1288	275.1289	-0.1	-0.36	7.0

Figure S34. HR-ESI-MS spectrum of compound **4**

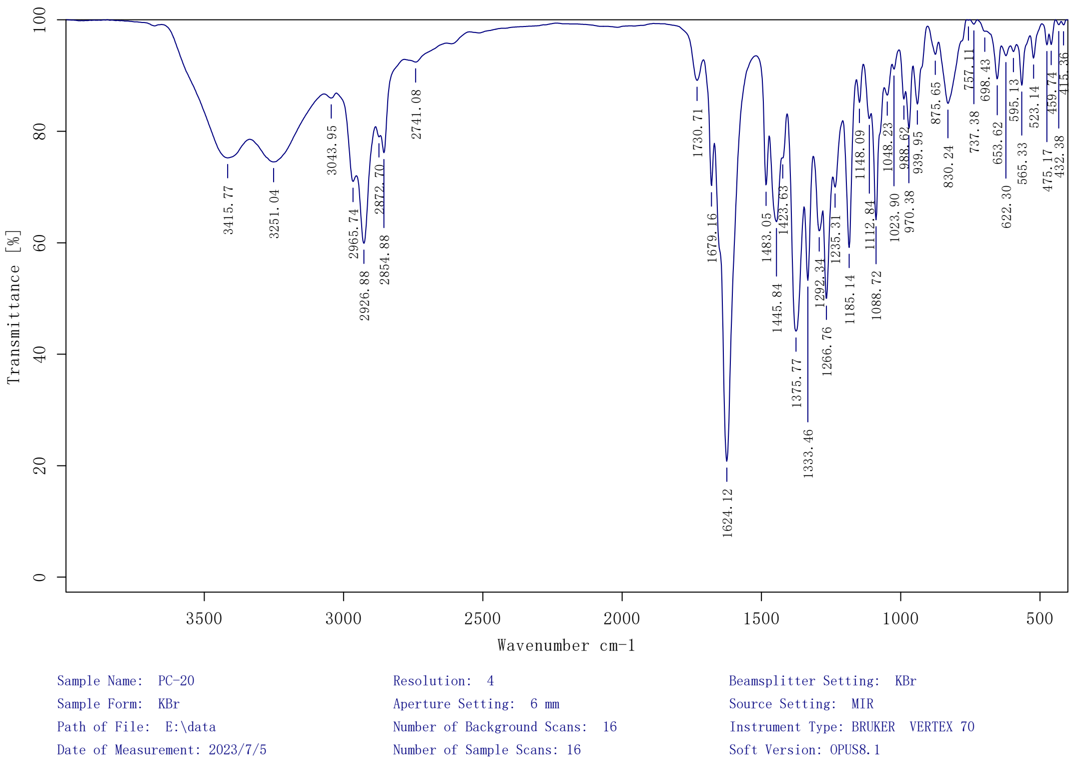

Figure S35. IR spectrum of compound **4**

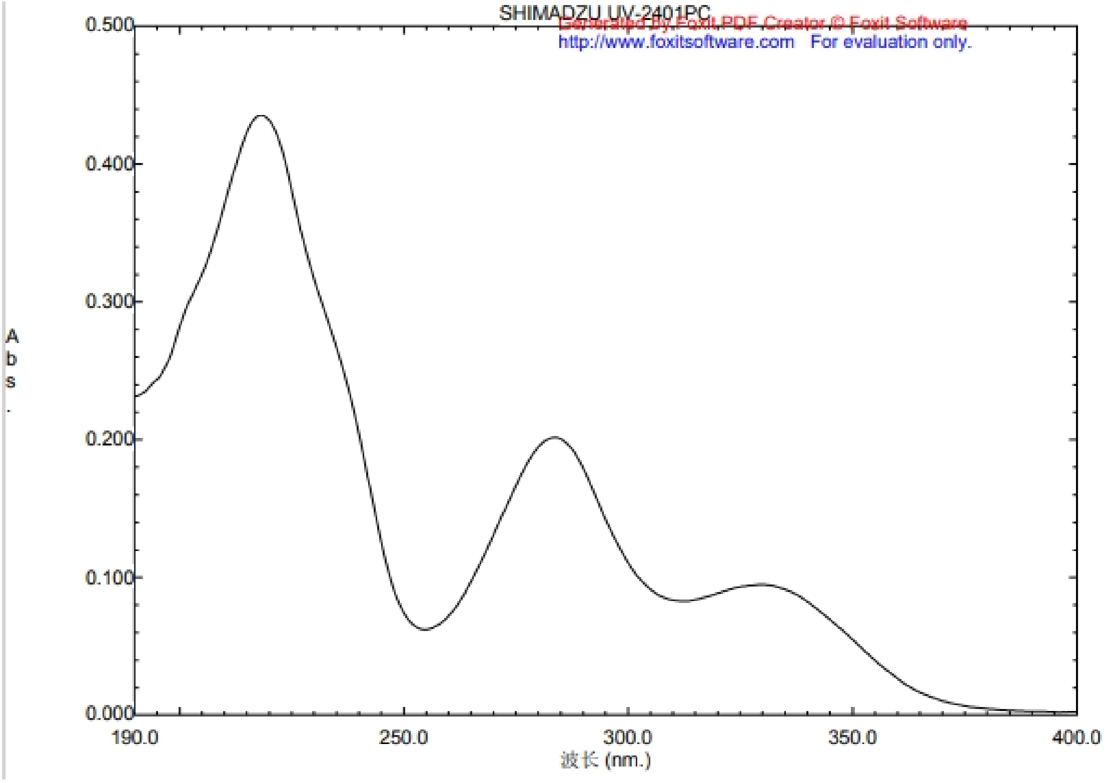

Figure S36. UV spectrum of compound **4**

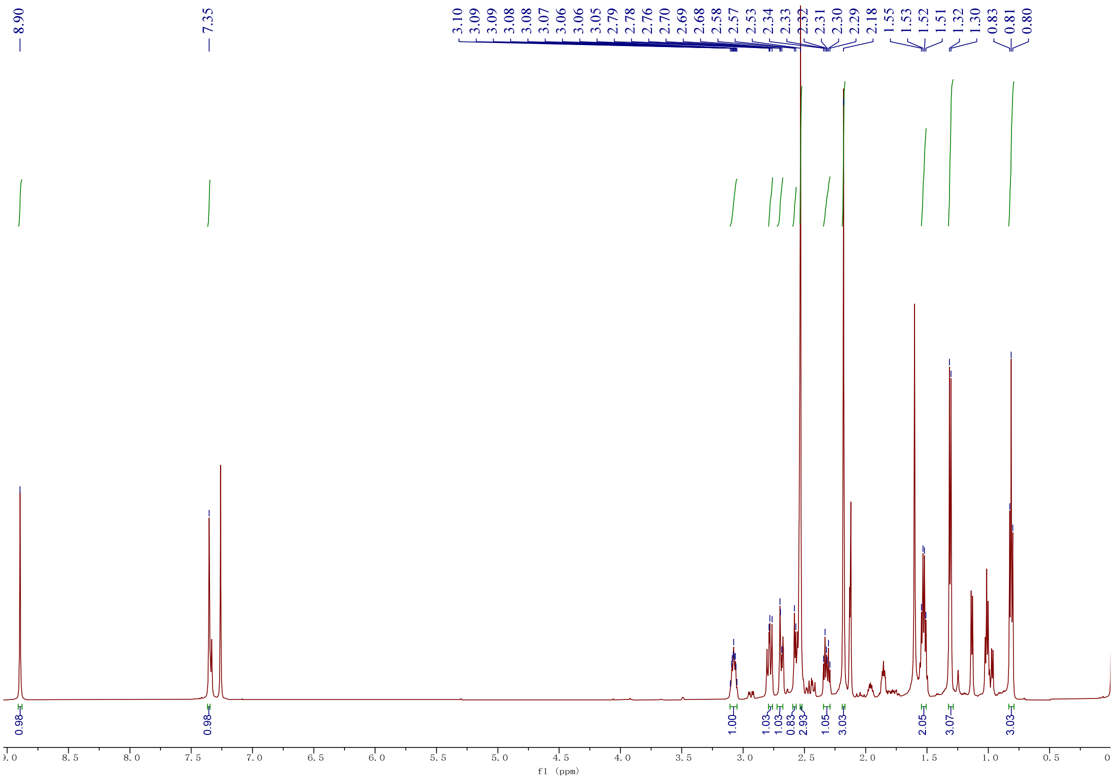

Figure S37. ^{1}H NMR spectrum (600MHz, $CDCl_3$) of **5**

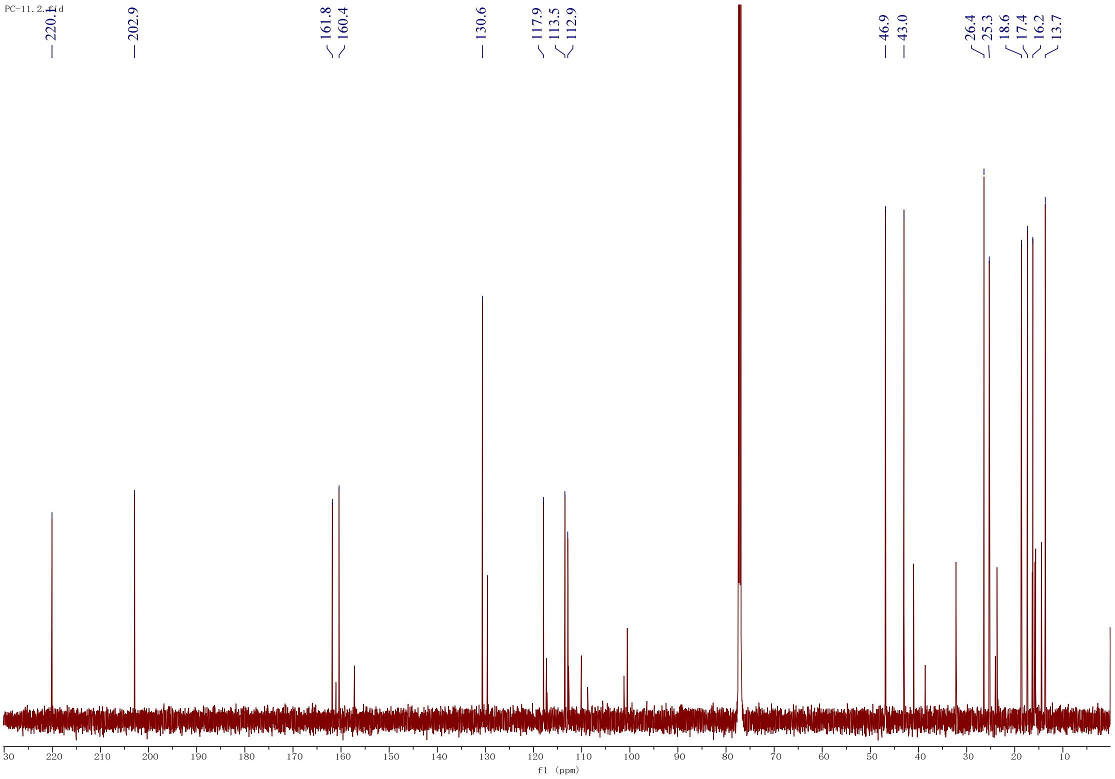

Figure S38. ^{13}C spectrum (150MHz, $CDCl_3$) of **5**

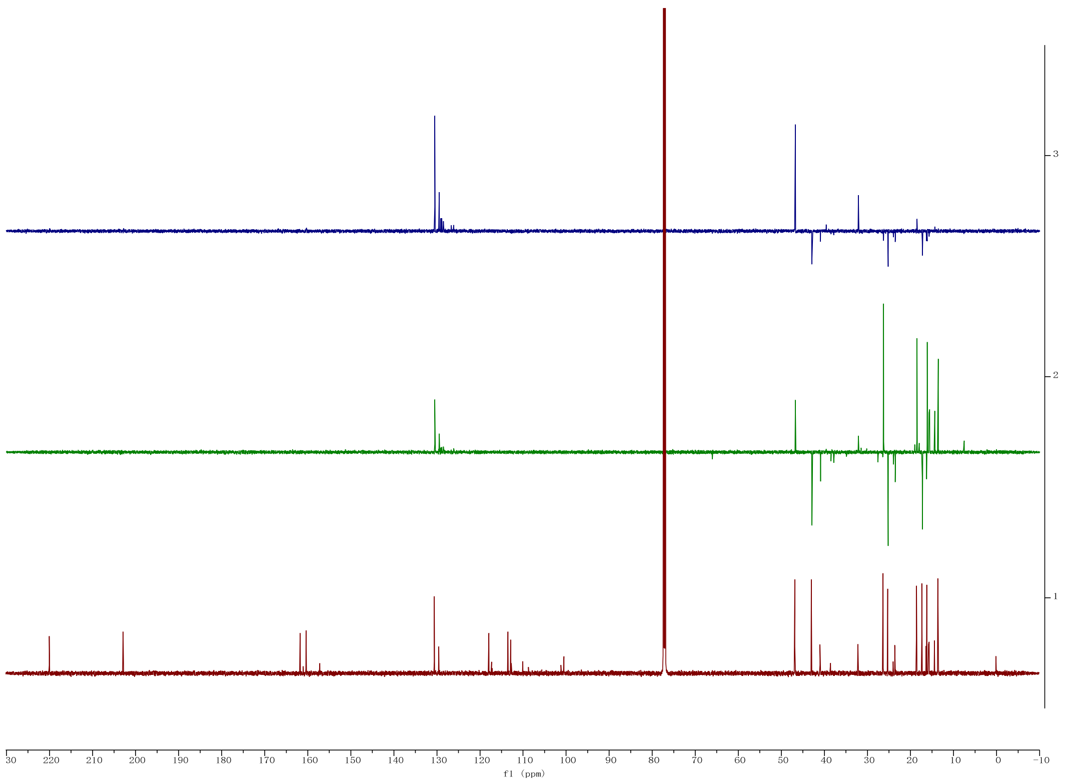

Figure S39. DEPT spectrum (150MHz, $CDCl_3$) of **5**

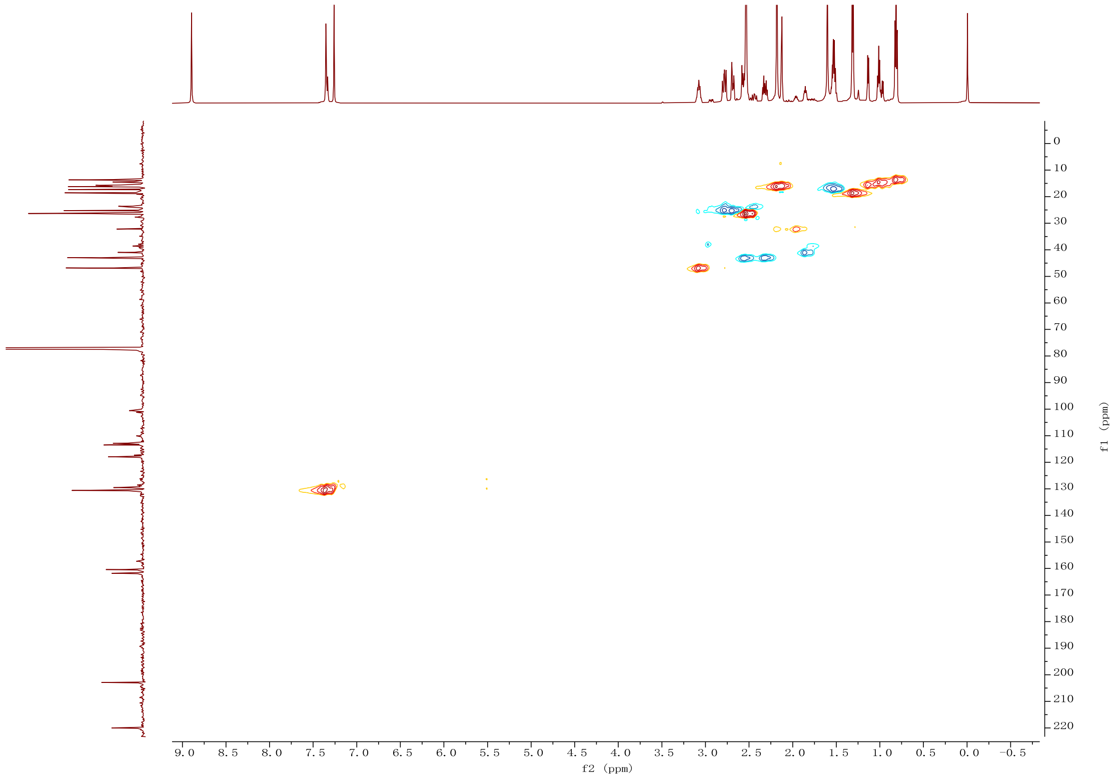

Figure S40. HSQC spectrum of **5**

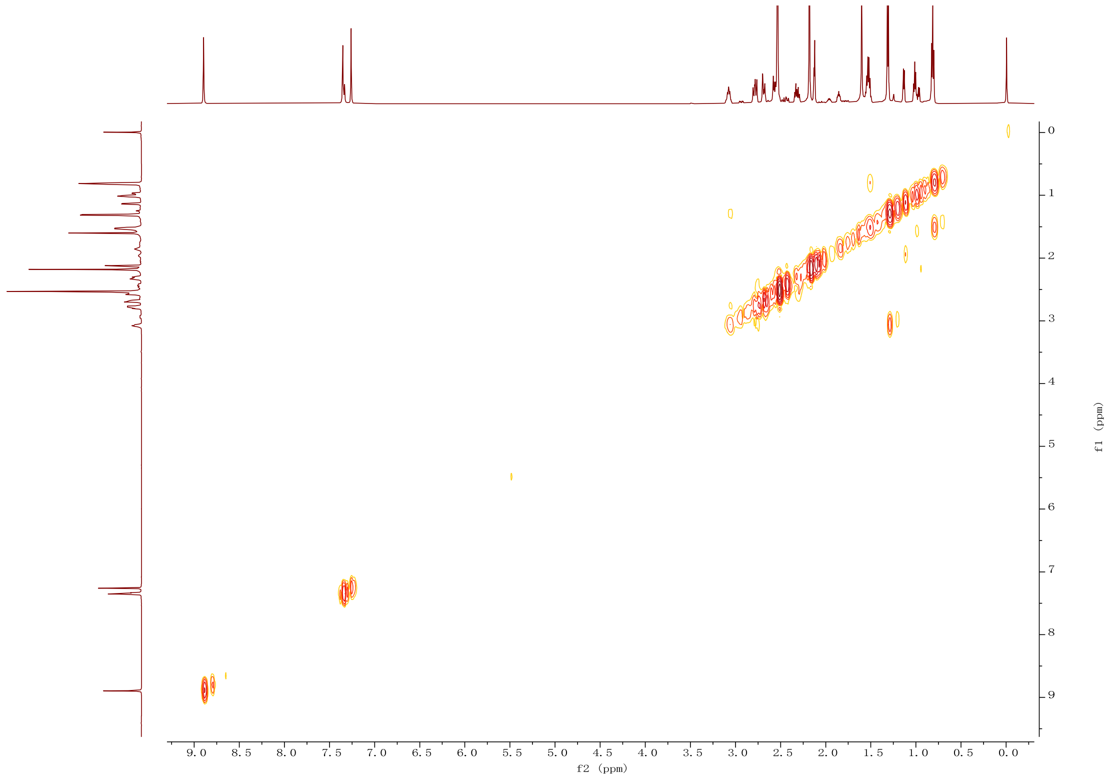

Figure S41. ^{1}H-^{1}H COSY spectrum of **5**

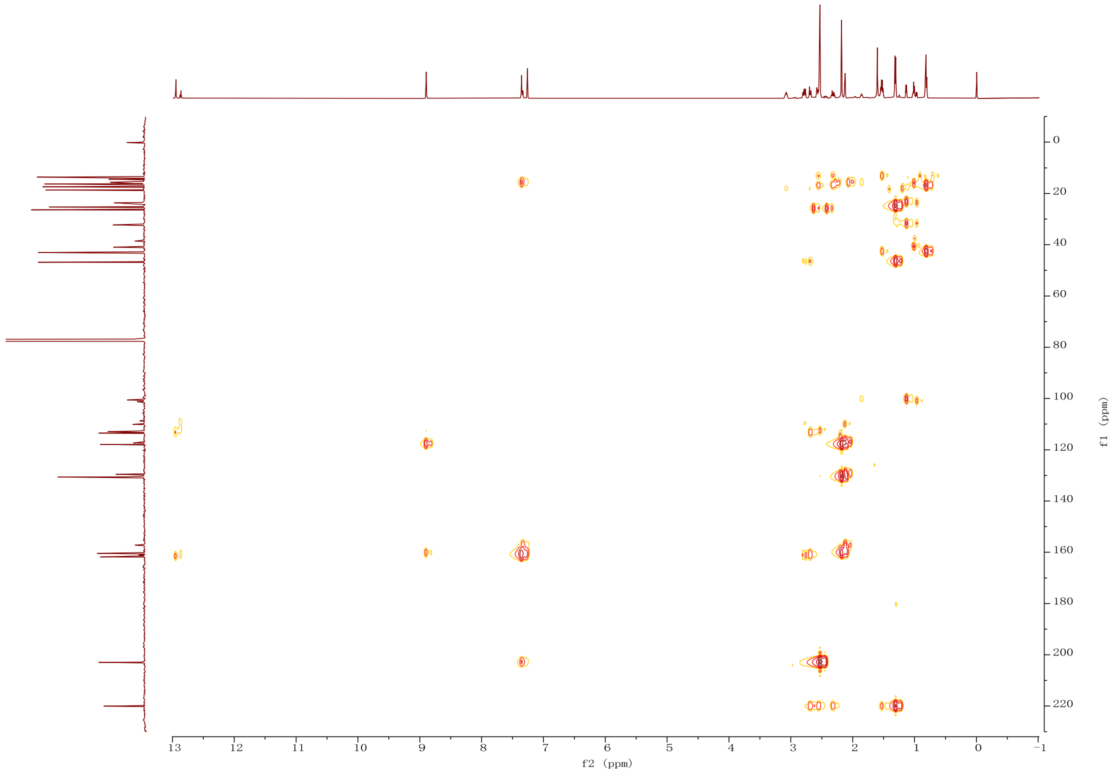

Figure S42. HMBC spectrum of **5**

Data File: E:\DATA\2023\0703\PC-24.lcd

Elmt	Val.	Min	Max	Elmt	Val.	Min	Max	Elmt	Val.	Min	Max	Elmt	Val.	Min	Max	Use Adduct
H	1	5	100	F	1	0	5	Cl	1	0	0	Ag	1	0	0	H
2H	1	0	0	Na	1	0	0	Co	2	0	0	I	3	0	5	
B	3	0	0	Mg	2	0	0	Cu	2	0	0	Ir	3	0	0	
C	4	5	100	Si	4	0	0	Se	2	0	0					
N	3	0	10	P	3	0	0	Br	1	0	0					
O	2	0	30	S	2	0	5	Pd	2	0	0					

Error Margin (ppm):	5	DBE Range:	not fixed	Electron Ions:	both
HC Ratio:	unlimited	Apply N Rule:	no	Use MSn Info:	yes
Max Isotopes:	all	Isotope RI (%):	1.00	Isotope Res:	10000
MSn Iso RI (%):	75.00	MSn Logic Mode:	OR	Max Results:	30

Event#: 2 MS(E-) Ret. Time : 0.613 -> 0.920 Scan# : 94 -> 140

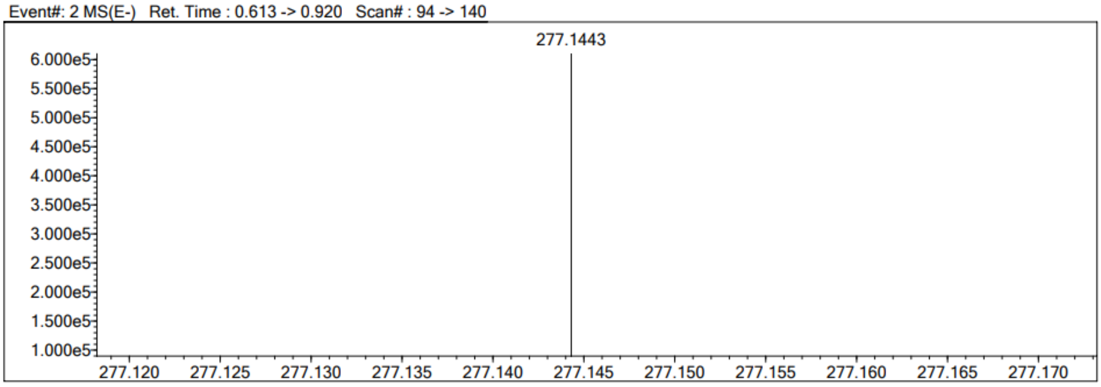

Measured region for 277.1443 m/z

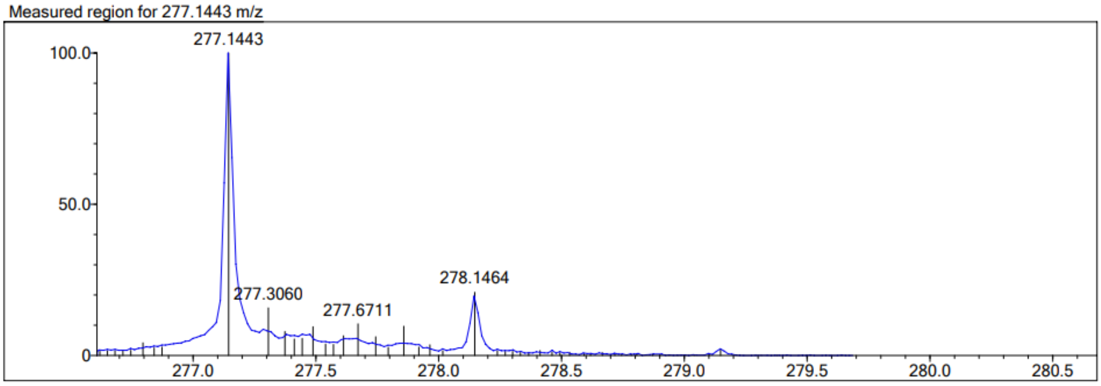

C16 H22 O4 [M-H]- : Predicted region for 277.1445 m/z

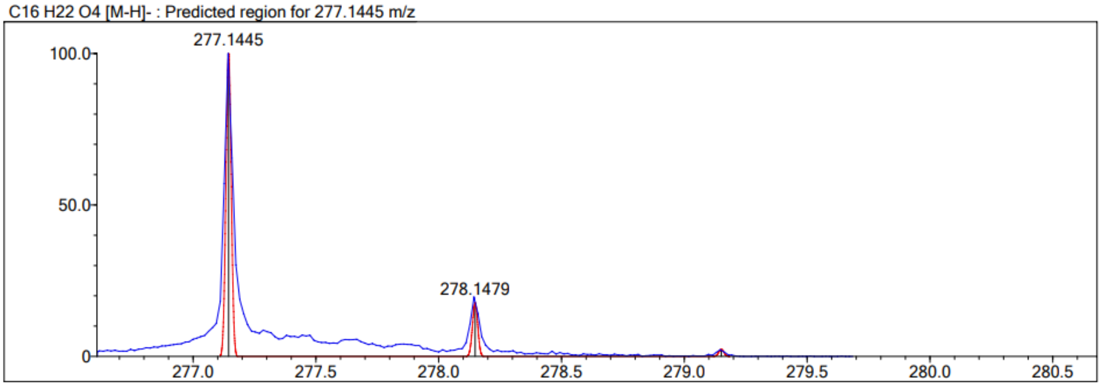

Formula (M)	Ion	Meas. m/z	Pred. m/z	Df. (mDa)	Df. (ppm)	DBE
C16 H22 O4	[M-H]-	277.1443	277.1445	-0.2	-0.72	6.0

Figure S43. HR-ESI-MS spectrum of compound **5**

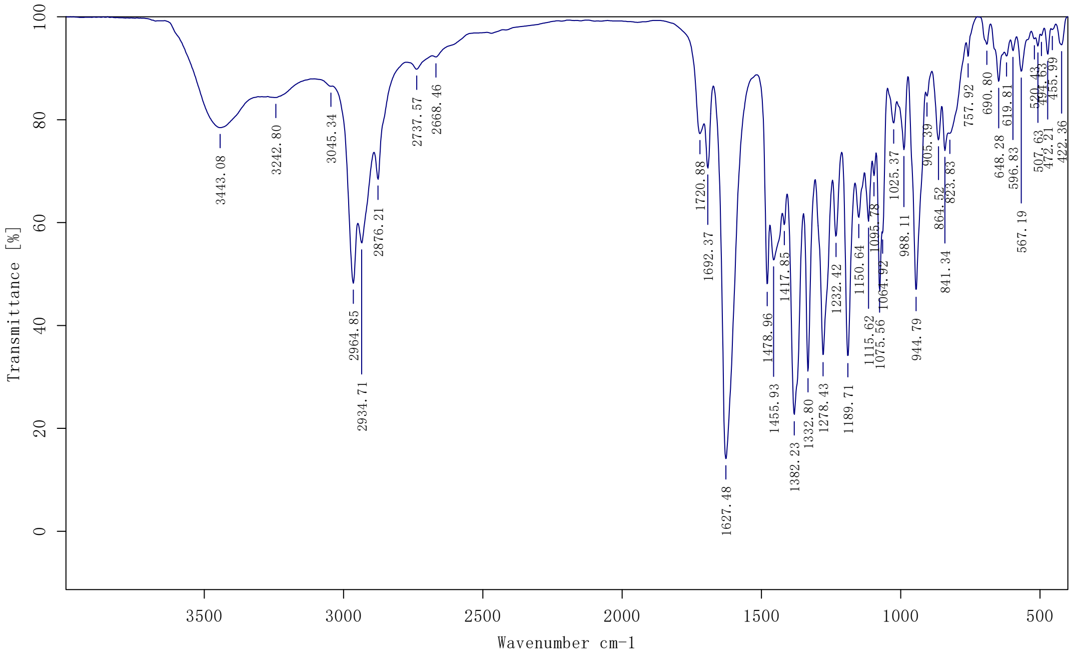

Sample Name: PC-24
Sample Form: KBr
Path of File: E:\data
Date of Measurement: 2023/7/5

Resolution: 4
Aperture Setting: 6 mm
Number of Background Scans: 16
Number of Sample Scans: 16

Beamsplitter Setting: KBr
Source Setting: MIR
Instrument Type: BRUKER VERTEX 70
Soft Version: OPUS8.1

Figure S44. IR spectrum of compound **5**

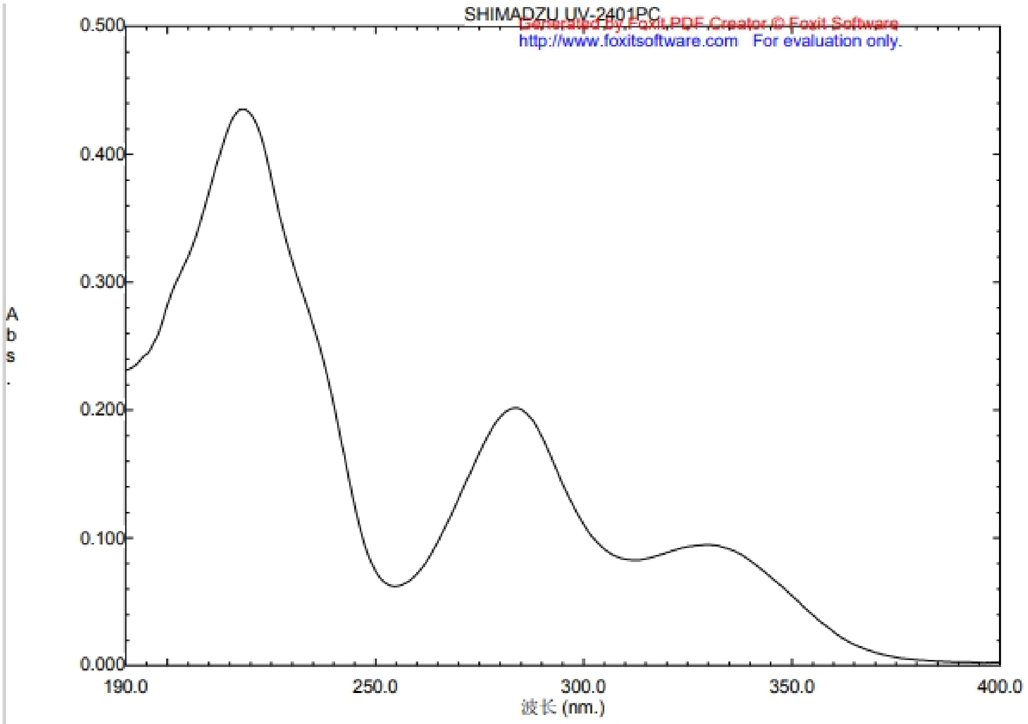

Figure S45. UV spectrum of compound **5**

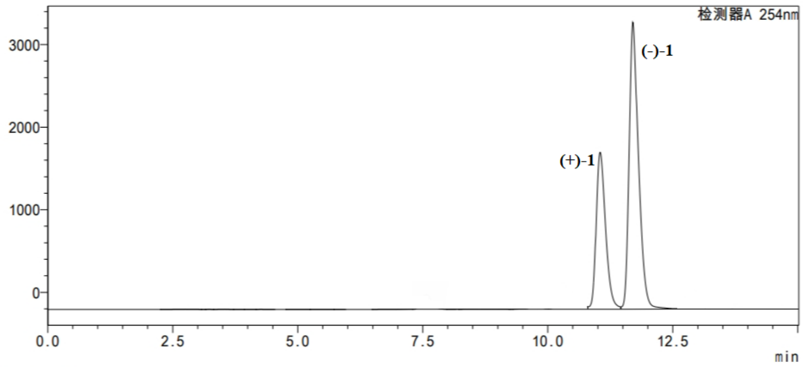

Figure S46. Chiral analysis and preparation of **(±)-1** (CHIRALCEL OJ-H, 5 μm, 4.6 mm × 250 mm; n-hexane/isopropanol = 90/10; flowing speed: 1 mL/min, 254nm).

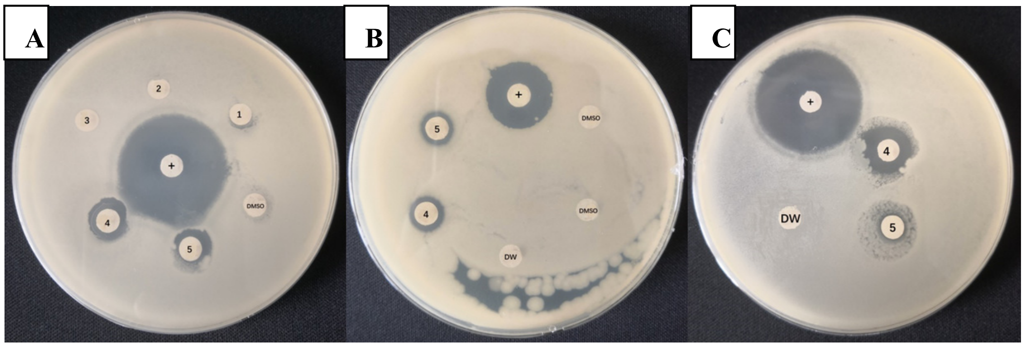

Figure S47. Initial evaluation of compounds 1-5 for their activity against *Shigella Castellani*. DW, deionized water; DMSO is used to eliminate solvent interference in experiments. ABC represents three repetitions.

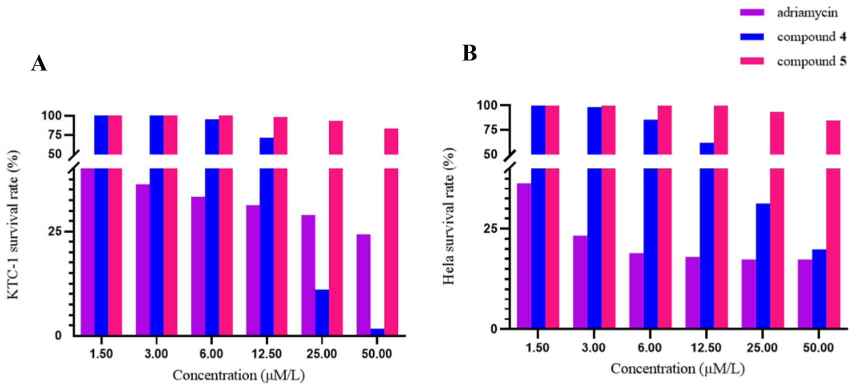

Figure S48. The inhibitory effect of compounds 4 and 5 on tumor cells KTC-1 and Hela. (A & B) were tested for cell viability using the CCK-8 method. The positive drug is adriamycin.
